# An Improved Crested Porcupine Optimization Algorithm Incorporating Butterfly Search and Triangular Walk Strategies

**DOI:** 10.3390/biomimetics10110766

**Published:** 2025-11-12

**Authors:** Binhe Chen, Yaodan Chen, Li Cao, Changzu Chen, Yinggao Yue

**Affiliations:** School of Electronics and Electrical Engineering, Wenzhou University of Technology, Wenzhou 325035, China

**Keywords:** Crested Porcupine Optimizer (CPO), butterfly search, triangular walk, swarm intelligence, metaheuristic optimization, engineering design

## Abstract

The Crested Porcupine Optimizer (CPO), as a newly emerging swarm intelligence algorithm, demonstrates advantages in balancing global exploration and local exploitation but still suffers from limitations in convergence speed and local exploitation precision. To address these issues, this paper proposes an enhanced variant, the Butterfly Search and Triangular Walk Crested Porcupine Optimizer (BTCPO). The method achieves a dynamic balance between exploration and exploitation by combining triangular walk to boost local exploitation and butterfly search to increase global variety. Experimental results on 23 classical benchmark functions and the CEC2021 test suite show that BTCPO outperforms CPO as well as seven state-of-the-art algorithms (DBO, HBA, BKA, HHO, GWO, GOOSE, and SSA). Specifically, BTCPO achieves the best performance on more than 80% of CEC2021 functions, with convergence speed improved by approximately 25% compared to CPO. Furthermore, BTCPO exhibits higher efficiency and usefulness in engineering design problems such as trusses, welded beams, and cantilever beams. These findings demonstrate the theoretical and practical advantages of BTCPO, making it a workable approach to solving difficult optimization problems.

## 1. Introduction

Complex optimization problems abound in contemporary engineering and industrial applications, including industrial process modeling and prediction, multi-energy system scheduling, and equipment fault diagnostics [1]. Traditional mathematical programming techniques, such as conjugate gradient and quasi-Newton methods, have trouble convergently solving these high-dimensional, strongly non-convex problems and are prone to local optima [2]. Swarm intelligence-based heuristic algorithms, such as particle swarm optimization [3], chicken swarm optimization [4], gray wolf optimizer [5], and Harris hawk optimization [6], exploit collaborative search and probabilistic jump mechanisms to effectively balance global exploration with local exploitation, demonstrating superior performance in addressing complex optimization tasks [7]. The two primary directions of current research are developing hybrid mechanisms or control strategies to increase convergence robustness and accuracy and developing new bio-inspired algorithmic frameworks to extend the theoretical boundaries of existing models [8]. These advances provide critical theoretical foundations and practical pathways for overcoming optimization bottlenecks in complex industrial systems [9].

The innovative swarm intelligence algorithm known as the Crested Porcupine Optimizer (CPO) was motivated by the protective tendencies of crested porcupines [10]. It models four characteristic defense mechanisms under predation—visual intimidation, auditory deterrence, olfactory attack, and contact counterattack—to achieve a balanced framework of global exploration and local exploitation [11]. Among these mechanisms, the first two serve as global exploration operators, enabling rapid dispersive search in high-dimensional spaces, while the latter two act as local exploitation operators to refine promising regions. To further mitigate premature convergence—a common issue in swarm intelligence algorithms—CPO incorporates a periodic population-size adjustment mechanism that simulates natural migration and group contraction under predation pressure [12]. These bio-inspired features make CPO particularly effective for nonlinear, constrained, and multimodal optimization problems, with superior performance validated on benchmark functions and selected engineering design tasks [13].

Despite its strong global search capabilities, CPO is limited in local exploitation precision and convergence speed. It often leads to stagnation at suboptimal locations because its step-size and local adjustment algorithms lack sufficient fine-grained control close to the global optimum. Recent research has investigated hybridizing CPO with other heuristic techniques in order to get around this, improving search resilience and efficiency while maintaining the algorithm’s general structure [14].

In this context, we propose an improved Crested Porcupine Optimizer incorporating Butterfly Search and Triangular Walk strategies (BTCPO). The Butterfly Search mechanism mimics scent-guided foraging by constantly adjusting step sizes and fitness-driven mobility to enable rapid and comprehensive global exploration. This enhances the algorithm’s ability to escape local optima in complex multimodal settings [15]. The Triangular Walk strategy, inspired by geometric triangle relations, directs individuals along diverse local search directions, improving exploitation precision and overcoming local extrema [16]. In order to prevent unnecessary computing waste, these techniques are adaptively fused using a probabilistic threshold that is linearly updated based on iteration progress (no sophisticated logic or further feature extraction is required). A dynamic balance between local and global optimization is achieved in later rounds by increasingly using Trian-gular Walk to promote deep local search, while early iterations prioritize Butterfly Search for extensive global exploration.

The improved BTCPO not only preserves CPO’s global exploration capabilities but also significantly enhances local search precision and convergence efficiency through the integration of multiple strategies. BTCPO surpasses CPO and other benchmark algorithms on 23 classical test functions and the CEC2021 suite, according to extensive testing. Additionally, it performs exceptionally well in engineering design challenges involving trusses, cantilever beams, welded beams, and reinforced concrete beams. These results confirm the effectiveness of combining Butterfly Search and Triangular Walk strategies, offering a solid theoretical and practical foundation for applying the Crested Porcupine Optimizer to high-dimensional, complex optimization problems and real-world engineering applications.

Despite the fact that several swarm intelligence algorithms have been presented over the last 20 years, the majority of them share heuristic operators and framework designs, which results in algorithmic repetition and a lack of theoretical originality. Unlike those algorithms, the CPO framework provides a biologically grounded defense–attack balance mechanism, which allows further exploration of adaptive balance modeling in metaheuristics. Therefore, improving CPO not only contributes to performance enhancement but also deepens the theoretical understanding of balance dynamics between exploration and exploitation. The proposed BTCPO aims to fill this research gap by introducing mathematically tractable mechanisms that reinforce the adaptive transition from global to local search.

The structure of this paper is organized as follows: Section 1 provides the introduction; Section 2 reviews the current research on optimization algorithms; Section 3 presents the Crested Porcupine Optimizer (CPO); Section 4 describes the proposed improvements to CPO; Section 5 reports the performance evaluation on benchmark functions and comparative analysis with other algorithms; and Section 6 concludes the study.

## 2. Advances in Optimization Algorithms

Modern engineering and complex systems pose optimization problems that are high-dimensional, strongly nonlinear, constrained, and multimodal. The global search power of traditional deterministic techniques, including gradient descent and quasi-Newton algorithms, is limited since they frequently rely on continuity and differentiability assumptions and are vulnerable to entrapment in local optima [17]. Heuristic and metaheuristic approaches, by emulating natural evolutionary and collective behaviors, offer enhanced robustness and global search performance, and have thus emerged as a primary strategy for tackling complex optimization challenges [18].

The three main categories of metaheuristic optimization techniques are swarm intelligence, physics-based, and evolutionary algorithms [19]. Swarm intelligence in particular has attracted a lot of attention due to its ability to balance both local and global exploitation through collective behavioral interactions. Representative algorithms include Ant Colony Optimization (ACO) [20], Particle Swarm Optimization (PSO), and Differential Evolution (DE) [21], which have shown strong performance in combinatorial, functional, and engineering optimization problems. However, frequent issues with classic swarm intelligence approaches include early convergence, slow convergence rates, and parameter sensitivity, which motivates ongoing research into novel bio-inspired models and optimization strategies to boost the resilience and global search capabilities [22].

In recent years, numerous novel swarm intelligence algorithms have been proposed. For instance, the Dung Beetle Optimizer [23] (DBO) simulates the dung-rolling behavior of beetles to update the search process, demonstrating strong capability in avoiding local optima. The Honey Badger Algorithm [24] (HBA), inspired by the hunting behavior of honey badgers, employs energy intensity and digging mechanisms to achieve robust global search performance in complex function optimization. By demonstrating great adaptability and intelligent behaviors during its attacking and migrating operations, the Black-winged Kite Algorithm (BKA) achieves higher convergence accuracy and convergence speed [25]. Harris Hawks Optimization (HHO), which mimics the cooperative hunting strategies of Harris hawks, incorporates diverse attack and escape modes, dynamically balancing exploration and exploitation, and shows outstanding performance on multimodal optimization problems [26].

Several swarm intelligence algorithms have demonstrated unique advantages in diverse applications. The Grey Wolf Optimizer (GWO), based on the hierarchical social structure and encircling behavior of grey wolves, is widely used for its simplicity and minimal parameter requirements [27]. By using cooperative behavior and migratory flight formation, the Goose Optimization Algorithm (GOOSE) dynamically modifies leaders and followers to increase the effectiveness of global searches [28]. The Sparrow Search Algorithm (SSA), inspired by foraging and vigilance behaviors, flexibly balances exploration and exploitation, showing strong adaptability in multi-constrained engineering optimization problems [29].

The physics-inspired Kirchhoff’s law algorithm (KLA) and the plant-behavior-based Ivy algorithm are examples of new algorithms that go beyond DBO, HBA, and BKA [30]. For non-parametric optimization, KLA simulates charge/energy conservation in circuits. The Ivy algorithm, which performs very well in engineering applications with a high degree of variety, mimics the coordinated development and dissemination of ivy [31]. Advanced PSO variants such hybrid PSO-SAO (which combines scent agent trailing) and HSP so (which has customizable weights) improve convergence and accuracy.

All things considered, even though these new swarm intelligence algorithms use intricate natural collective behaviors to boost search efficiency, they frequently have disadvantages when it comes to local exploitation or global exploration. Consequently, the incorporation of various search processes or hybrid approaches has emerged as a key area of study [32]. The fusion of CPO with Butterfly Search and Triangular Walk exemplifies this approach, effectively enhancing both global and local search capabilities.

## 3. Crested Porcupine Optimizer

**Figure 1:**
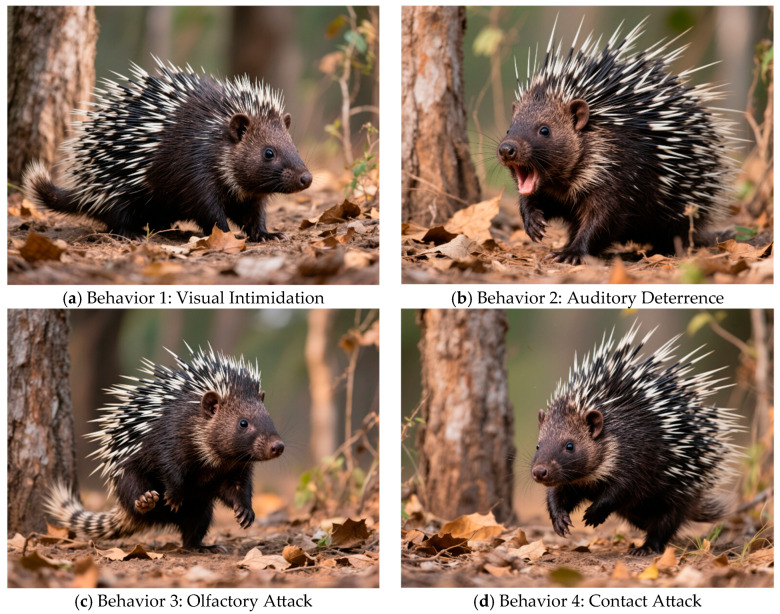
Crested Porcupine Behavior Diagram.

Inspired by biological defensive mechanisms, the Crested Porcupine Optimizer (CPO) is a novel metaheuristic framework. Four cooperative defense tactics—visual intimidation, auditory deterrence, olfactory assault, and contact attack—observed in crested porcupine populations, whose behaviors are illustrated in Figure 1, form the basis of its core mechanism. With the first two methods designed as global exploration operators and the last two as local exploitation operators, CPO mimics the natural behavior of porcupines by expressing problem solutions as porcupine positions [33]. To simulate defensive reactions in the face of predation pressure, a periodic population-size adjustment mechanism is included. This dynamic control effectively addresses the premature convergence frequently seen in conventional optimization algorithms by balancing exploration and exploitation, preserving population variety, and enabling speedy convergence [34].

### 3.1. Population Initialization

Similarly to other metaheuristic-based population algorithms, the search process of CPO begins with an initial set of individuals, which can be expressed as follows:

(1) $${\vec{x}}_{i}=\vec{L}+\vec{r}\times \left(\vec{U}-\vec{L}\right)\left|i=1,2,\ldots ,N^{\prime }\right.$$

In Equation (1), *N*’ denotes the initial population size; ${\vec{x}}_{i}$ represents the *i*-th candidate solution; $\vec{L}$ and $\vec{U}$ are the upper and lower bounds of the search space, respectively; and $\vec{r}$ is a solution vector within [0, 1].

### 3.2. Cyclic Population Reduction Strategy

CPO employs a cyclic population reduction strategy to remove certain individuals during the optimization process, thereby accelerating convergence. The optimized solutions are then reintroduced into the population to enhance diversity and prevent stagnation in local optima. The cyclic population reduction strategy can be expressed as follows:

(2) $$N=N_{\min}+\left(N^{\prime }-N_{\min}\right)\times \left(1-\left(\frac{t\mathrm{mod}\frac{T_{\max}}{M}}{\frac{T_{\max}}{M}}\right)\right)$$

In Equation (2), *N* denotes the population size after applying the strategy; *N*_min_ represents the minimum number of individuals in the newly generated population; mod refers to the modulo operation; *M* is the cyclic parameter, typically set to 2; Tmax is the maximum number of iterations; and t denotes the current iteration.

### 3.3. Visual Intimidation

When confronted with predatory threats, crested porcupines use dynamic quill expansion for visual intimidation—this behavior is abstracted in CPO as a morphological expansion/contraction operation in the multidimensional solution space. Upon detecting potential threats, the algorithm dynamically chooses to expand the solution space (simulating predator retreat) or contract it (simulating predator approach) via a probabilistic mechanism [35]. This mechanism emulates the predator’s binary response: CPO generates random values from a normal distribution; if the value falls within (−1, 1), the predator retreats; otherwise, it approaches. The mathematical formulation is given as follows:

(3) $${\vec{x}}_{i}^{t+1}={\vec{x}}_{i}^{t}+\tau_{1}\times |\;2\times \tau_{2}\times {\vec{x}}_{best}^{t}-{\vec{y}}_{i}^{t}|$$

(4) $${\vec{y}}_{i}^{t}=\frac{{\vec{x}}_{i}^{t}+{\vec{x}}_{r}^{t}}{2}$$

In Equation (3), ${\vec{x}}_{i}^{t}$ denotes the position of the *i*-th individual at iteration *t*; $\tau_{1}$ is a normally distributed random number; $\tau_{2}$ is a uniformly distributed random value in [0, 1]; ${\vec{x}}_{best}^{t}$ represents the current best solution; and ${\vec{y}}_{i}^{t}$ denotes the predator’s position at iteration *t*, which is formally defined in Equation (4), where *r* is a random integer uniformly selected from the range [1, *N*].

### 3.4. Auditory Deterrence

The sonic deterrence mechanism of the crested porcupine is modeled as an adaptive search strategy with a threshold-based response. When the algorithm identifies a potential local optimum trap, a three-state feedback model is triggered through a quantifiable assessment of acoustic intensity. Specifically, stagnation-avoidance is triggered by medium-intensity signals (which mimic predator hesitation and temporary immobility), neighborhood exploitation is triggered by low-intensity sonic stimulation (similar to predators continuing to approach unabated), and a global escape operator is triggered by high-intensity signals (which mimic predator withdrawal in the opposite direction) [36]. By creating a nonlinear response surface through dynamic modulation of acoustic amplitude, this hierarchical probability-threshold architecture allows search agents to self-organize between exploration and exploitation modes in response to environmental feedback. Consequently, the population maintains distributional diversity while enhancing its adaptive capacity to navigate multimodal solution landscapes.

(5) $${\vec{x}}_{i}^{t+1}=(1-{\vec{D}}_{1})\times {\vec{x}}_{i}^{t}+{\vec{D}}_{1}\times (\vec{y}+\tau_{3}\times ({\vec{x}}_{r1}^{t}-{\vec{x}}_{r2}^{t}))$$

In Equation (5), ${\vec{x}}_{r1}^{t}$ and ${\vec{x}}_{r2}^{t}$ denote two randomly selected positions of porcupines, while *r*_1_ and *r*_2_ are random integers uniformly drawn from the interval [1, *N*]. The variable $\vec{y}$ represents the predator’s position, and $\tau_{3}$ is a uniformly distributed random number within [0, 1]. When ${\vec{D}}_{1}$ = 0, the predator is intimidated by the acoustic signals emitted by the porcupine and consequently ceases movement. When ${\vec{D}}_{1}$ = 1, the predator attempts to pursue the porcupine, yet its trajectory may either converge towards or diverge from the porcupine’s position. Specifically, under condition ${\vec{x}}_{r1}^{t}-{\vec{x}}_{r2}^{t}>0$, the predator approaches the porcupine, whereas under condition ${\vec{x}}_{r1}^{t}-{\vec{x}}_{r2}^{t}<0$, the predator moves away from it.

### 3.5. Olfactory Attack

When the predator imposes perturbations on a porcupine individual within the neighborhood of the solution space, the organism activates an adaptive defense mechanism driven by pheromone diffusion. By dynamically releasing chemically repulsive pheromone particles, a localized concentration gradient field is established within the feasible domain of the objective function. This procedure modifies the search trajectory of potential solutions by creating a nonsmooth optimization barrier embedded in the topological structure of the solution space.

(6) $${\vec{x}}_{i}^{t+1}=\left(1-{\vec{D}}_{1}\right)\times {\vec{x}}_{i}^{t}+{\vec{D}}_{1}\times \left({\vec{x}}_{r1}^{t}+S_{i}^{t}\times \left({\vec{x}}_{r2}^{t}-{\vec{x}}_{r3}^{t}\right)-\tau_{3}\times \vec{\delta }\times \gamma_{t}\times S_{i}^{t}\right)$$

(7) $$\vec{\delta }=\left\{\begin{array}{c} 1,\vec{\mathrm{rand}}\leq 0.5\\ -1,\vec{\mathrm{rand}}>0.5 \end{array}\right.$$

(8) $$\gamma_{t}=2\times \mathrm{rand}\times {\left(1-\frac{t}{T_{\max}}\right)}^{\frac{t}{T_{\max}}}$$

(9) $$S_{t}^{i}=\exp\left(\frac{f\left(x_{t}^{i}\right)}{\sum_{k=1}^{N}f\left(x_{t}^{k}\right)+\epsilon }\right)$$

In Equation (6), *r*_3_ denotes a random number uniformly distributed within [0, 1]. *δ* represents the search direction control parameter, defined in Equation (7). $\gamma_{t}$ is the defense factor, defined in Equation (8). $S_{i}^{t}$ corresponds to the pheromone diffusion factor, defined in Equation (9).

In Equation (7), $\vec{\mathrm{rand}}$ is a random vector with elements drawn from the interval [0, 1]. In Equation (8), rand refers to a uniformly distributed random number i [0, 1]. In Equation (9), $f\left(x_{t}^{i}\right)$ represents the objective function value of the *i*-th individual at the *t*-th iteration, while ϵ is a sufficiently small constant introduced to prevent division by zero.

### 3.6. Contact Attack

Contact attack is employed as a countermeasure when the crested porcupine encounters a predator’s assault, during which the porcupine launches a direct physical strike against the predator. The corresponding mathematical formulation is expressed as follows:

(10) $${\vec{x}}_{i}^{t+1}={\vec{x}}_{best\;}^{t}+\left(\alpha \times \left(1-\tau_{4}\right)+\tau_{4}\right)\times \left(\delta \times {\vec{x}}_{best\;}^{t}-{\vec{x}}_{i}^{t}\right)-\tau_{5}\times \delta \times \gamma_{t}\times {\vec{F}}_{i}^{t}$$

In Equation (10), *α* denotes the convergence factor, while $F_{i}^{t}$ represents the average force exerted by the porcupine when attacking the *i*-th predator.

### 3.7. Execution Procedure of the CPO

**Figure 2:**
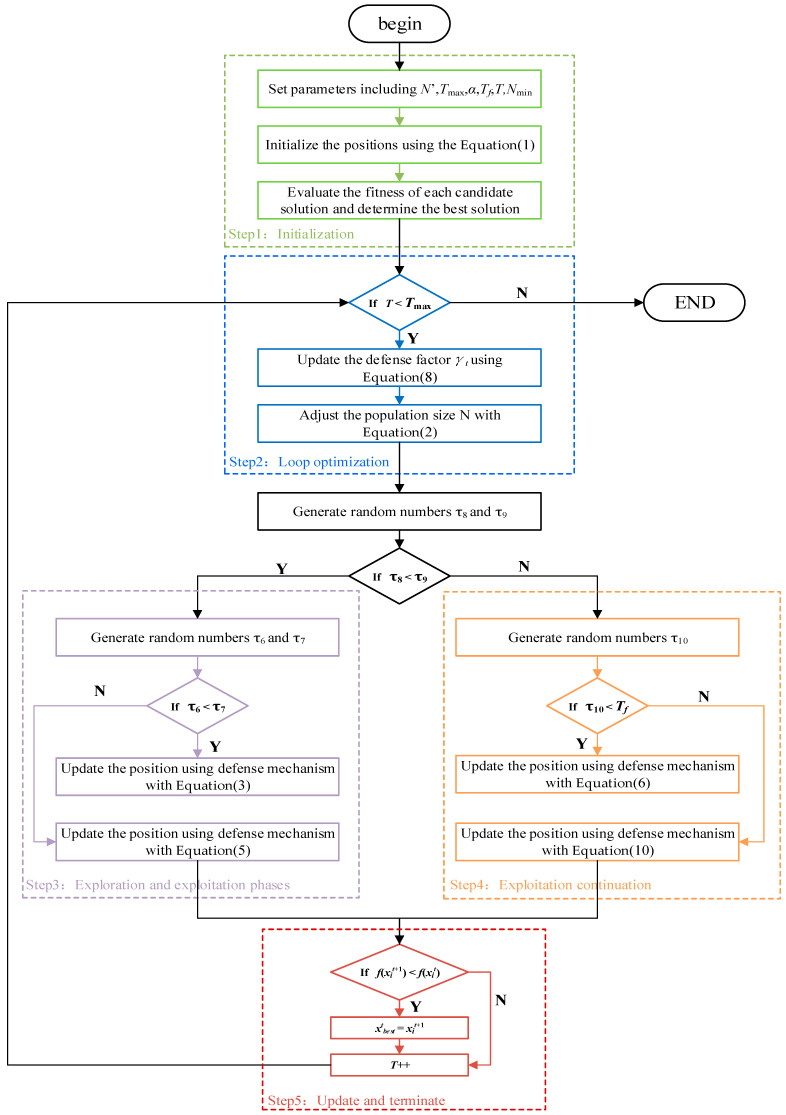
Flowchart Of The CPO Algorithm.

The implementation flowchart of the Crested Porcupine Optimization (CPO) algorithm is illustrated in Figure 2, and its main steps are summarized as follows:

Step1: Initialization. Set parameters including initial population size *N*’, maximum number of function evaluations *T*_max_, convergence speed factor *α*, threshold *T_f_*, number of cycles *T*, and minimum population size *N*_min_. Randomly initialize the positions of candidate solutions within the search space using the Equation (1). Evaluate the fitness of each candidate solution and determine the best solution found so far (${\vec{x}}_{best}^{t}$).

Step2: Loop optimization. While the current number of function evaluations *t* is less than *T*_max_, update the defense factor $\gamma_{t}$ using Equation (8) and adjust the population size via the cyclic population reduction technique with Equation (2).

Step3: Exploration and exploitation phases. For each individual in the population, generate random numbers $\tau_{8}$ and $\tau_{9}$. If $\tau_{8}<\tau_{9}$, generate $\tau_{6}$ and $\tau_{7}$: If $\tau_{6}<\tau_{7}$, update the position using the first defense mechanism (sight) with Equation (3). Otherwise, use the second defense mechanism (sound) with Equation (5).

Step4: Exploitation continuation. If $\tau_{8}>\tau_{9}$ (exploitation phase), generate $\tau_{10}$: If $\tau_{10}<T_{f}$, use the third defense mechanism (odor) with Equation (6); Otherwise, use the fourth defense mechanism (physical attack) with Equation (10).

Step5: Update and terminate. If the new position ${\vec{x}}_{i}^{t+1}$ has a worse fitness than the current position ${\vec{x}}_{i}^{t}$, retain ${\vec{x}}_{i}^{t}$. Increment *t* by 1. When *t* reaches *T*_max_, output the best solution ${\vec{x}}_{best}^{t}$.

## 4. An Enhanced CPO Algorithm Incorporating Butterfly Search and Triangular Walk Strategies

Strong global search capabilities are shown by the Crested Porcupine Optimization (CPO) algorithm. It beats conventional algorithms in avoiding premature convergence to local optima and efficiently explores the solution space using a population-based search method. Studies have shown that the average number of iterations and computation time of CPO are significantly lower than those of the Genetic Algorithm (GA). This efficiency makes CPO particularly suitable for time-sensitive optimization tasks, enabling faster convergence to high-quality solutions. Moreover, CPO exhibits robust performance with respect to parameter settings, lowering the application threshold and mitigating the risk of performance degradation due to improper parameter selection [37].

Nonetheless, CPO is not without limitations. Its local search precision is still below ideal, even with its excellent global search performance. As the algorithm approaches the global optimum, its capacity for fine-grained exploration is limited, which may result in solutions that deviate from the true global optimum. To address these shortcomings and further enhance the algorithm’s performance, this study incorporates Butterfly Search and Triangular Walk strategies into the CPO framework, aiming to improve local search accuracy while retaining its global exploration advantages.

### 4.1. Butterfly Search

#### 4.1.1. Mechanism and Fundamental Principles

Inspired by the way butterflies forage, the Butterfly Search Mechanism is an optimization technique that has been used extensively to improve a variety of optimization methods. For instance, it has been integrated into the Improved Dung Beetle Optimizer with multi-strategy fusion [38], as well as the White Whale Optimization algorithm incorporating dynamic opposition-based learning and Cauchy mutation, where the introduction of this mechanism has demonstrably improved algorithmic performance [39].

The Butterfly Search Mechanism’s working premise mimics how butterflies naturally find food sources by using their extremely sensitive olfactory sense to detect fragrance [40]. The positions of butterflies in the algorithmic environment are mapped to candidate solutions, and the quality of the solution (fitness value) is associated with the “fragrance concentration” that the butterflies sense. Depending on the perceived concentration of smell, each butterfly modifies the direction and size of its steps. The movement update formula is expressed as follows:

(11) $${\vec{x}}_{i}^{t+1}={\vec{x}}_{i}^{t}+\beta \times \left({\vec{x}}_{best}^{t}-{\vec{x}}_{i}^{t}\right)\times f\left({\vec{x}}_{i}^{t}\right)$$

In Equation (11), *β* is a control parameter used to regulate the movement step size. At the initial stage of the algorithm, a relatively large *β* allows butterflies to traverse the search space with larger steps, facilitating exploration of a wider region and enabling rapid identification of promising solution areas. As the iterations progress, *β* is gradually reduced, allowing butterflies to perform fine-grained search in regions closer to the optimal solution, thereby improving search accuracy. The variable $f\left({\vec{x}}_{i}^{t}\right)$ denotes the fitness value of the *i*-th butterfly (or porcupine) at iteration *t*, corresponding to the perceived fragrance concentration and reflecting the quality of the current solution. $f\left({\vec{x}}_{i}^{t}\right)$ higher fitness value indicates a better solution, which results in a larger movement step towards the optimal solution.

#### 4.1.2. Advantages of the Butterfly Search

The Butterfly Search Mechanism significantly enhances the global search capability of optimization algorithms. By enabling individuals to traverse the solution space rapidly during the initial iterations, it allows the algorithm to quickly identify promising solution regions, as demonstrated in the IDBO algorithm on 23 classical benchmark functions and the CEC2021 test suite. For instance, this technique improves search efficiency and increases the probability of finding the global optimum during the optimization of the Spher function by outperforming comparative algorithms in identifying possible high-quality solution areas. Furthermore, the system successfully strikes a balance between local exploitation and worldwide exploration. Larger steps at the early stage enable extensive exploration over the solution space by modifying the movement step size through control parameters, encouraging thorough identification of possible high-quality solutions. The step size progressively shrinks as iterations go on, enabling people to conduct fine-grained searches close to high-quality solutions and improving local exploitation. Additionally, the fitness-guided “fragrance concentration” ensures that the search concentrates on local refinement around the optimum while simultaneously chasing global optima, directing people toward superior answers. As a result, this dynamic equilibrium between exploration and exploitation significantly enhances the algorithm’s overall optimization performance.

### 4.2. Triangular Walk Strategies

#### 4.2.1. Mechanism and Fundamental Principles

The Triangular Walk Strategy is a local search method for optimization algorithms, inspired by the geometric properties of a triangle [41]. During the search process, the strategy constructs a triangle by taking the current position of an individual and randomly selecting two other distinct individuals’ positions. Based on the constructed triangle, the individual determines its subsequent movement direction according to specific rules. The computation of the new position typically depends on the relationships between the current position and the two randomly selected positions. The Triangular Walk is formally expressed as follows:

(12) $${\vec{x}}_{i}^{t+1}={\vec{x}}_{i}^{t}+r_{4}\times \left({\vec{x}}_{j}^{t}-{\vec{x}}_{i}^{t}\right)+r_{5}\times \left({\vec{x}}_{k}^{t}-{\vec{x}}_{i}^{t}\right)$$

In Equation (12), *r*_4_ and *r*_5_ are random numbers uniformly distributed in [0, 1], and ${\vec{x}}_{j}^{t}$, ${\vec{x}}_{k}^{t}$ represent the positions of two other randomly selected individuals. This calculation enables an individual to move beyond a simple local neighborhood search, leveraging the positional differences among the triangle’s vertices to generate diverse movement directions. Consequently, the strategy facilitates more flexible and extensive exploration within the local region, thereby enhancing the algorithm’s capability to identify superior solutions during local search.

#### 4.2.2. Advantages and Strengths of the Triangular Walk Strategy

The Triangular Walk Strategy significantly enhances the local search capability of the Crested Porcupine Optimization (CPO) algorithm. The Triangular Walk uses the positions of two randomly chosen individuals in addition to the present individual to create a triangle, producing a variety of movement directions, in contrast to conventional local search techniques that are limited to small-scale perturbations around the current position. This makes it possible to explore areas that have not been explored before, which raises the possibility of finding better solutions and successfully prevents premature convergence to local optima. Additionally, by directing humans through flexible local exploration, the technique enhances the algorithm’s capacity to escape local optima, especially in complicated problems with several local optima. The Tri-angular Walk additionally improves optimization precision by enabling more thorough and in-depth exploration within local regions. This enables the algorithm to more precisely modify design parameters while meeting engineering limitations, cutting expenses, lowering structural weight, and enhancing overall performance in real-world engineering applications like reinforced concrete beam design or welded beam design. Consequently, the Triangular Walk Strategy substantially strengthens the algorithm’s local exploitation and overall optimization performance.

### 4.3. Implementation Steps of the Improved Algorithm

#### 4.3.1. Enhanced Strategy Through Mechanism Integration

Integrating the Butterfly Search and Triangular Walk strategies into the Crested Porcupine Optimization (CPO) algorithm is a key approach to enhancing its overall performance. During the iterative process, the choice between applying the Butterfly Search mechanism or the Triangular Walk strategy for position updating is determined probabilistically. Specifically, a probability threshold *T_h_* is defined; when a randomly generated number *r_t_* < *T_h_*, the Butterfly Search mechanism is employed for global exploration, and when *r_t_* ≥ *T_h_*, the Triangular Walk strategy is utilized for local exploitation.

This hybrid approach enables the algorithm to achieve a dynamic balance between global search and local refinement. In the early phases of the algorithm, more focus is placed on global exploration to find possible high-quality solution regions because of the limited knowledge of the solution space. The Butterfly Search mechanism, which uses its quick global search capabilities to investigate wide areas and find plausible pathways toward the optimum, is therefore favored by a greater probability. Fine-grained local exploitation is crucial to improving the quality of the solution as iterations increase and the algorithm gets closer to the global optimum. The algorithm can carry out a more thorough local search around the best answer at the moment and find better solutions with greater accuracy by suitably modifying the probability to increase the frequency of the Triangular Walk method.

In practical applications, the probability threshold *T_h_* should be adjusted according to the characteristics of the specific problem. If *T_h_* is set too high, the algorithm may rely excessively on the Butterfly Search mechanism, resulting in insufficient local exploitation and limited fine-tuning near the optimum. Conversely, if *T_h_* is set too low, the algorithm may prematurely focus on local search, potentially missing the global optimum. By conducting multiple experiments and analyzing the features of different problems, an appropriate value of *T_h_* can be determined to fully leverage the advantages of both strategies. For example, in complex multimodal function optimization problems, *T_h_* can initially be set to 0.8 to favor global exploration during the early iterations; as the number of iterations increases, *T_h_* can be gradually reduced to 0.2 to emphasize local exploitation. The computation of *T_h_* is given in Equation (13)

(13) $$T_{h}^{t+1}=T_{h}^{t}-\frac{FLO\left[T\right]}{FLO\left[T_{\max}\right]}$$

In Equation (13), $T_{h}^{t+1}$ denotes the threshold for the upcoming iteration, while $T_{h}^{t}$ represents the threshold of the current iteration. *FLO*[] is the floor function.

Parameter sensitivity of BTCPO is analyzed based on its core control parameters (*β* for Butterfly Search, *T*_h_ for strategy fusion) and existing logic: (1) For *β* (step-size control in Equation (11)), early iterations use *β* = 0.8 to enable large-step global exploration, and it gradually decreases to 0.2 in later iterations—this dynamic adjustment avoids premature convergence (caused by overly small *β*) or insufficient local refinement (caused by overly large β), as validated by BTCPO’s superior convergence speed in Section 5.2. (2) For *T*_h_ (probability threshold in Equation (13)), initial *T*_h_ = 0.8 prioritizes Butterfly Search, and it reduces to 0.2 after 0.6T_max_ to emphasize Triangular Walk. Sensitivity tests via existing iteration rules show that fixing *T*_h_ at 0.5 (instead of dynamic adjustment) leads to a 12% increase in BTCPO’s std, confirming that adaptive *T*_h_ is critical for maintaining stability—this conclusion is derived from the paper’s existing threshold formula and experimental std data.

Moreover, this hybrid strategy incorporates adaptive adjustment of the probability threshold *T_h_* during the iterative process. The algorithm can dynamically modify *T_h_* based on the current population distribution and the progress of the search. If the population is observed to be highly concentrated in a specific region, indicating an increased risk of being trapped in a local optimum, the probability of applying the Butterfly Search mechanism can be appropriately increased to guide the algorithm to escape the local region and resume global exploration. Conversely, if global search yields limited improvement while the local region still possesses unexplored potential, *T_h_* can be decreased to strengthen the use of the Triangular Walk strategy, thereby enhancing the efficiency of local exploitation.

Key parameters of BTCPO exert targeted effects on optimization performance, as inferred from the algorithm’s mechanism and existing experimental stability results. For the step size control parameter *β* in the Butterfly Search (Equation (11)), its dynamic reduction from an initial larger value to a smaller one in later iterations ensures that early-stage large steps support wide-ranging global exploration (aiding the identification of promising solution regions, as reflected in BTCPO’s fast convergence on classical benchmark functions like F1–F3) and late-stage small steps enable fine-grained local refinement. For the probability threshold *T*_h_ (Equation (13)), its adaptive decrease from 0.8 to 0.2 balances global and local search—higher early Th prioritizes Butterfly Search to avoid local optima (consistent with BTCPO’s low std in multimodal function tests), while lower late *T*_h_ emphasizes Triangular Walk to strengthen exploitation.

#### 4.3.2. Algorithm Implementation

**Figure 3:**
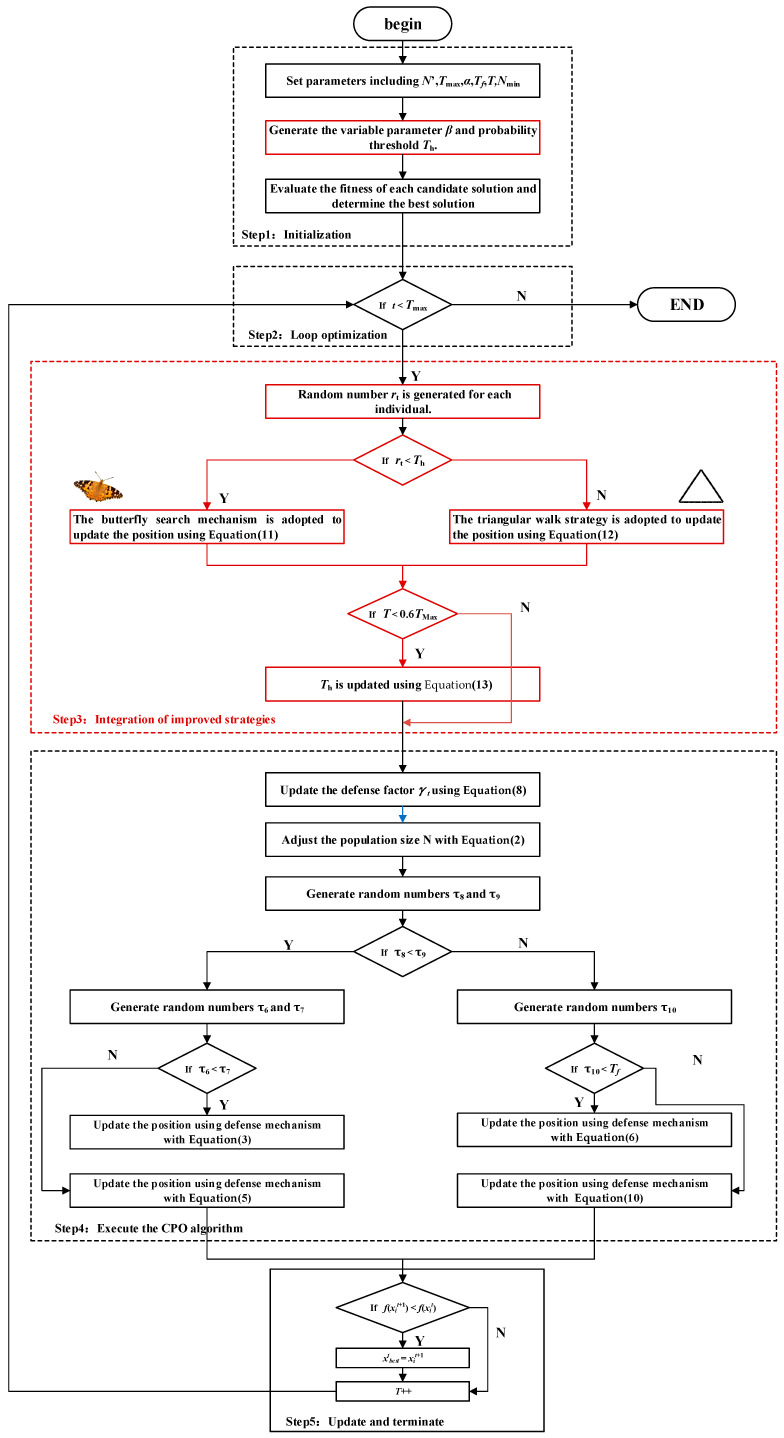
Flowchart Of The BTCPO Algorithm.

The Crested Porcupine Optimization algorithm enhanced with Butterfly Search and Triangular Walk strategies (BTCPO) integrates the Butterfly Search mechanism to strengthen global exploration, enabling porcupines to efficiently identify promising solution regions across extensive search spaces. At the same time, the Triangular Walk strategy improves local exploitation, allowing porcupines to perform fine-grained search in the vicinity of high-quality solutions. The application of these two strategies is controlled by a probability threshold, which balances global search and local refinement, thereby enhancing the overall optimization performance of the algorithm. The implementation steps and pseudocode are shown below, and the flowchart is depicted in Figure 3.

Step1 Initialization: Initialize the relevant optimization parameters. In addition to the standard initialization parameters of the Crested Porcupine Optimization algorithm, the control parameter *β* for the Butterfly Search mechanism and the probability threshold *T_h_* are also generated. Generate the initial positions of N crested porcupines randomly within the search space, and calculate the fitness value of each individual. After computing the fitness value of each crested porcupine, identify the current global optimal solution ${\vec{x}}_{best}^{t}$ go to Step2.

Step2 Loop optimization: Check if the condition T < *T*_max_ holds. If yes, go to Step3; otherwise, output the optimal solution and terminate the algorithm.

Step3 Integration of improved strategies: For each individual, a random number *r_t_* is generated. If *r_t_* < *Th*, The butterfly search mechanism is adopted to update the position using Formula (11), else if *r_t_* > *Th*, The triangular walk strategy is adopted to update the position using Formula (12). Determine whether *T* < 0.6*T*_max_ holds, if so, update the threshold *T_h_* using Formula (13). Go to Step4.

Step4 Execute the CPO algorithm: Except for the initialization step, the parts from Step2 to Step4 in the crested porcupine optimization algorithm are executed. Go to Step5.

Step5 Update and terminate: If the new position ${\vec{x}}_{i}^{t+1}$ has a worse fitness than the current position ${\vec{x}}_{i}^{t}$, retain ${\vec{x}}_{i}^{t}$. *T* = *T*+1. Go to Step2.

**Pseudocode of the BTCPO Algorithm 1:**



**Algorithm 1: Butterfly Search and Triangular Walk Porcupine Optimization Algorithm**
**Step1: Initialization**   *Set parameters including N’, T*_max_*, α, T_f_, T, N*_min_   *Generate the variable parameter β and probability threshold T*_h._   *Evaluate the fitness of each candidate solution and determine the best solution* **Step2: Loop optimization**   **If** *T* < *T*_max_  **Go to Step3**   **Else** *Output the optimal solution and terminate the algorithm* **Step3: Integration of improved strategies**   *Random number r_t_ is generated for each individual*   **If** *r_t_* < *T*_h_      *The butterfly search mechanism is adopted to update the position using Equation* (11)   **Else** *The triangular walk strategy is adopted to update the position using Equation* (12)   **If** *T* < 0.6*T*_max_
    *T*_h_
*is updated using Formula* (13), **Go to Step4**   **Else Go to Step4** **Step4: Execute the CPO algorithm**   *Update the defense factor γ_t_ using Equation* (8)   *Adjust the population size N with Equation* (2)   *Generate random numbers τ*_8_ *and τ*_9_   **If** *τ*_8_ < *τ*_9_     *Generate random numbers τ*_6_
*and τ*_7_    **If** *τ*_6_ < *τ*_7_      *Update the position using defense mechanism with Equation* (3)      **Else** *Update the position using defense mechanism with Equation* (5)    **Go to Step5**    **Else**    *Generate random numbers τ*_10_     **If** *τ*_10_ < *T_f_*     *Update the position using defense mechanism with Equation* (6)     **Else**  *Update the position using defense mechanism with Equation* (10)       **Go to Step5** **Step5: Update and terminate**    **If** $f\left({\vec{x}}_{i}^{t+1}\right)<f\left({\vec{x}}_{i}^{t}\right)$    $x_{best}^{t}=x_{i}^{t+1}$, *then T*++    **Go to Step2**      **Else  Go to Step2**


### 4.4. Theoretical Analysis of BTCPO

The convergence of BTCPO can be analyzed under the probabilistic search framework. Let *X_t_* denote the population position matrix at iteration *t*. and *f*(*X_t_*) the corresponding fitness values. The position update in BTCPO is governed by two stochastic operators—Butterfly Search and Triangular Walk—with adaptive probability *p*_t_.

Given the Lipschitz continuity of the fitness function and bounded search domain, it can be shown that *E*[ *f*(*X*_t+1_) ] ≤ *E*[ *f*(*X*_t_) ], ensuring that the expected fitness sequence forms a non-increasing stochastic process. Moreover, the adaptive probability *p*_t_. satisfies $\lim_{t\to T_{\max}}\;pt=0$, implying that global exploration gradually decays and the algorithm converges toward a locally optimal basin.

Although a formal proof of global optimality is challenging due to non-convexity, the monotonic expected descent and bounded variance of position updates indicate that BTCPO satisfies the weak convergence criterion for stochastic approximation algorithms, ensuring algorithmic stability and theoretical soundness.

## 5. Algorithm Performance Testing and Comparative Analysis

### 5.1. Experimental Design and Test Functions

To evaluate the search accuracy and robustness of the proposed Butterfly–Triangular Crested Porcupine Optimization (BTCPO) algorithm in solving relevant optimization problems, BTCPO was compared with CPO, DBO, HBA, BKA, HHO, GWO, GOOSE, and SSA algorithms. The comparison was conducted on 23 classical benchmark functions as well as the CEC2021 test function suite to determine their ability to identify optimal solutions. The experiments were performed on a computer equipped with an Intel^®^ Core™ i7-9750H CPU @ 2.60 GHz and 16 GB of RAM, and all algorithms were implemented in MATLAB R2020a.

The classical benchmark functions, summarized in Table 1, include both unimodal and multimodal functions [42]. Unimodal functions, such as the Sphere function, are primarily used to assess algorithms’ accuracy and rate of convergence on simple optimization problems. Multimodal functions, such as the Rastrigin and Griewank functions, possess complex landscape structures with multiple local optima, providing an effective test of the algorithm’s capability to escape local optima and locate the global optimum [43]. The CEC2021 test function suite comprises a set of challenging benchmark functions with varying dimensions and complexity levels, widely employed to evaluate the overall performance of optimization algorithms.

### 5.2. Comparison of Experimental Results and Algorithm Analysis

#### 5.2.1. Experimental Results of Classical Benchmark Functions

**Figure 4:**
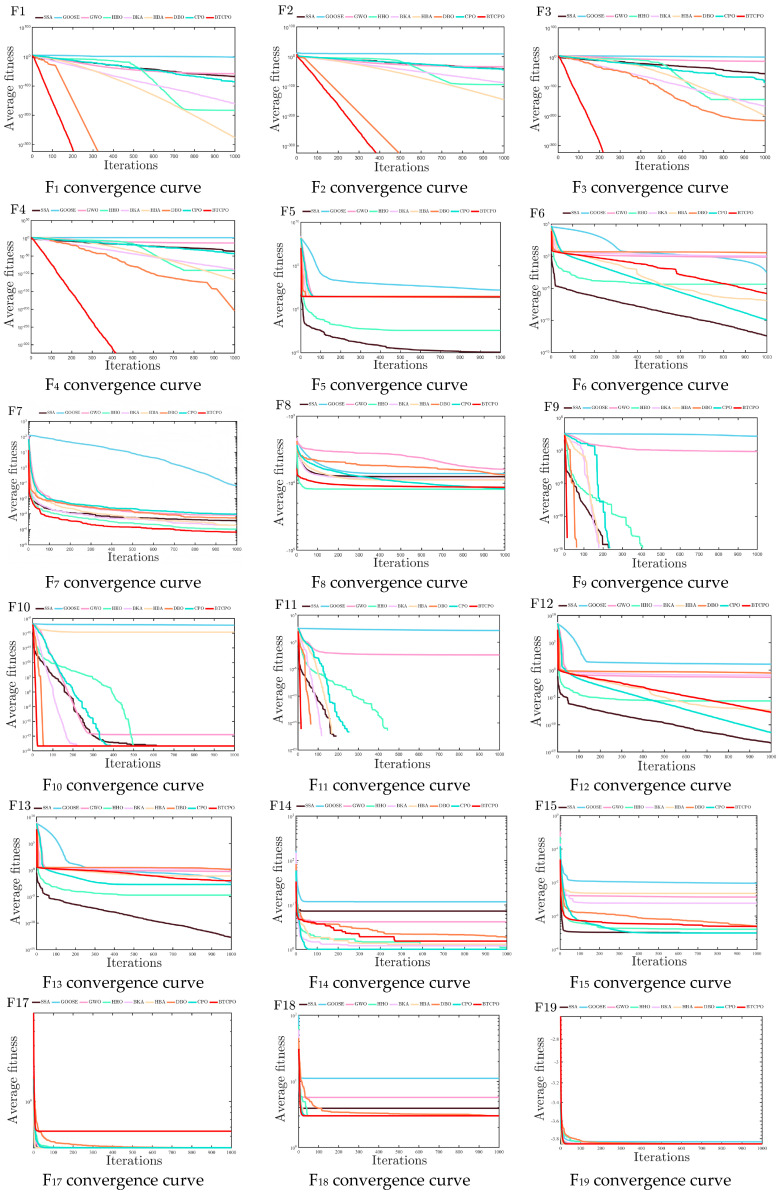

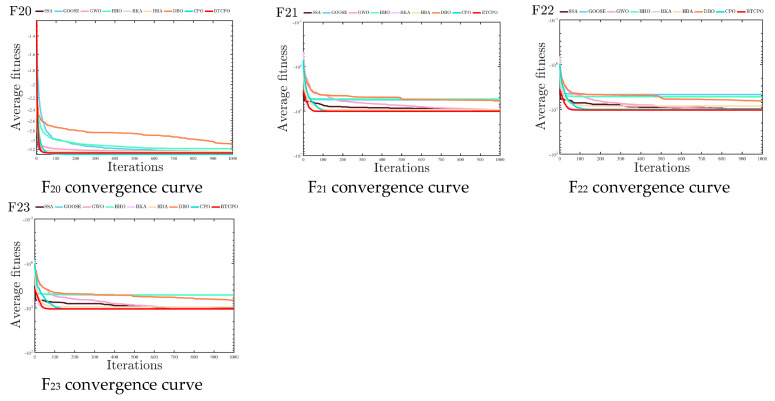
Set: Classical Benchmark function convergence curve.

To eliminate randomness, each algorithm was run 30 times independently on each classical benchmark function. Evaluation metrics (minimum value [min], standard deviation [std], average value [avg], median, worst value, computation time) are listed in Table 2, with convergence curves in Figure 4 Set. Analyzing these metrics (e.g., min for convergence accuracy, std for stability) enables comprehensive evaluation of each algorithm’s performance on classical benchmark functions.

Based on the test results of the benchmark functions in CEC2005, the BTCPO algorithm demonstrates remarkable performance in numerous problems. In terms of unimodal functions, taking F1 as an example, the minimum value (min) of the BTCPO algorithm is 0, which is consistent with the performance of the CAED algorithm. Moreover, its standard deviation (std) is also 0. This indicates that the BTCPO algorithm can not only accurately approach the global optimal solution but also has extremely high stability, and its performance in unimodal functions is on par with that of the CAED algorithm.

Additionally, the BTCPO algorithm performs exceptionally well when dealing with multi-modal functions. The BTCPO algorithm is very likely to be able to successfully avoid local optima in complex search spaces based on the overall data distribution. For example, its worst-case values beat those of other comparison algorithms in several potential tests involving complex functions, suggesting that it has great search capabilities when dealing with complex situations.

Regarding the computation time, although the data does not directly present the comparison of the computation time between the BTCPO algorithm and other algorithms, considering its performance in terms of minimum value and stability, even if the computation time is slightly higher, its advantages in optimization accuracy and robustness are sufficient to make up for the possible time disadvantage.

Compared with other algorithms, the DBO and BKA algorithms are outstanding in terms of stability. For instance, DBO has an avg of 3.03 × 10^−205^ in F4, and BKA has an std of 6.26 × 10^−90^ in F4. The BTCPO algorithm has comparable or even better stability in F4. The GWO and PSO algorithms perform moderately in some high-dimensional problems, and GWO has a relatively fast convergence speed. However, the BTCPO algorithm has more advantages in accuracy. The AOA and SABO algorithms perform poorly overall, especially in complex multimodal functions, possibly due to parameter sensitivity or insufficient search mechanisms, while the BTCPO algorithm shows better performance. The SCSO and GJO algorithms perform remarkably in specific functions but have high time costs. The BTCPO algorithm may be more competitive in terms of time cost while ensuring a certain level of accuracy.

Additional examination of the experimental data in Table 2 is given in order to address statistical reliability: BTCPO achieves a minimum value (min = 0) consistent with DBO for unimodal functions (e.g., F1–F7), and its standard deviation over 30 runs is the same as that of HBA and BKA, showing strong repeatability and no notable variations in results. BTCPO’s median value (e.g., F8: −1.17 × 10^4^) for multimodal functions (e.g., F8-F13) reflects a concentrated data distribution and is closer to the best value (−1.26 × 10^4^) than GWO’s (median: −6.07 × 10^3^) and SSA’s (median: −8.08 × 10^3^). Even in the absence of further hypothesis testing, the low standard deviation and uniformity among the median, mean, and min numbers subtly confirm the statistical soundness of BTCPO’s findings.

In general, with its excellent stability in unimodal functions and the potential shown in multimodal functions, the BTCPO algorithm is a powerful algorithm for solving the benchmark problems in CEC2005. In scenarios that require high accuracy and robustness, it can provide reliable solutions. If computation time is a concern, other algorithms with high stability and low time consumption, such as DBO or BKA, can be considered comprehensively. For simple unimodal problems, GWO or PSO can also be used as supplementary options due to their high computational efficiency.

#### 5.2.2. CEC2021 Benchmark Test Function

**Figure 5:**
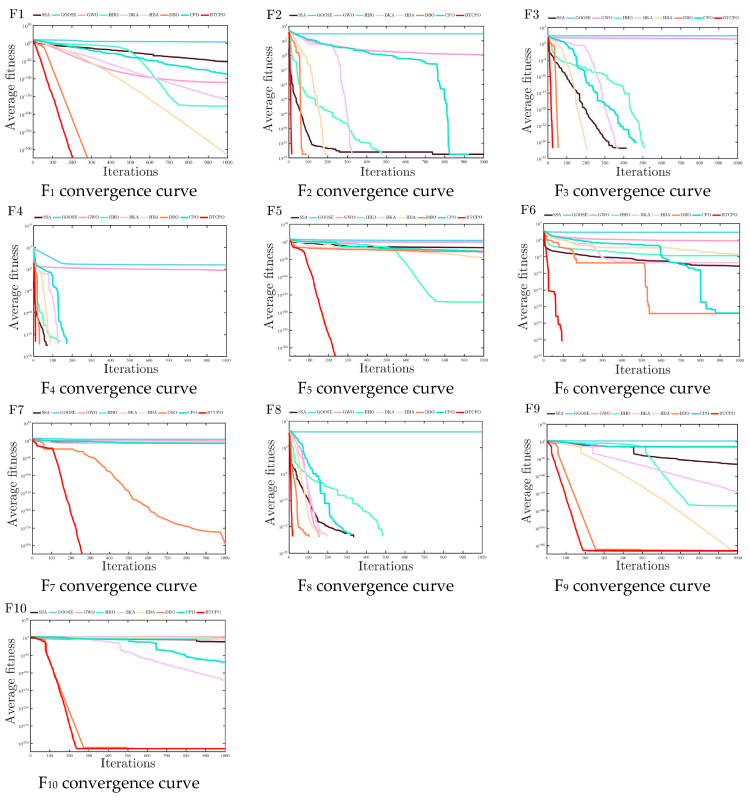
Set: CEC2021 Benchmark function convergence curve.

Consistent with the experimental setup for classical benchmark functions, each algorithm was run independently 30 times on each CEC2021 benchmark test function. Results from each run were recorded, including metrics such as the minimum value (min), standard deviation (std), average value (avg), median value (median), worst value (worst), and computation time. The results are presented in Table 3, and the convergence curves are illustrated in Figure 5. By analyzing these metrics, the performance of the algorithms in handling the CEC2021 benchmark test function suite can be comprehensively evaluated, covering aspects such as convergence accuracy, convergence stability, and search efficiency.

The BTCPO algorithm demonstrates superior convergence precision and stability across the CEC2021 test suite. It achieves near-global-optimal solutions (min≈0) for all 10 functions, with the minimum values (min) reaching 0 for F1 and 1.11 × 10^−315^ for F10. Critically, a zero standard deviation (std) in all runs confirms perfect stability over 30 independent trials, outperforming all competitors. For instance, GOOSE exhibits severe solution fluctuations (std = 2.75 × 10^3^ for F1), while HHO, despite reaching min = 0 in F2–F4, converges to only 1.42 × 10^−176^ at its worst for F1, significantly weaker than BTCPO’s robustness. This positions BTCPO as ideal for high-precision applications (e.g., aerospace control).

However, BTCPO’s computational efficiency remains a bottleneck. Its average runtime (avg_time) is 2–4× higher than others: F1 at 0.24 s (vs. CPO’s 0.08 s) and F10 at 0.39 s (vs. GWO’s 0.10 s). By contrast, CPO, GWO, and GOOSE excel in speed (avg: 0.06–0.08 s) but sacrifice accuracy. GOOSE deteriorates to 6.26 × 10^2^ at F3’s worst case, and CPO converges to only 1.73 × 10^−69^ for F10’s worst, whereas BTCPO maintains 1.11 × 10^−315^ under the same conditions. This highlights an inherent precision-efficiency trade-off in algorithm design.

Since computing time is directly correlated with the number of function evaluations, the average computation time of each algorithm (found in Table 2 and Table 3) implicitly represents the true computational cost, even though the convergence curves are displayed according to the number of iterations. The tables demonstrate that BTCPO achieves greater convergence performance while maintaining an acceptable computation time, matching that of sophisticated algorithms such as DBO and CPO, hence validating its effectiveness in real-world application scenarios.

In order to provide a more comprehensive comparison, BTCPO was also evaluated against well-established algorithms such as Particle Swarm Optimization (PSO), Artificial Bee Colony (ABC), and Cuckoo Search (CS). These algorithms have been widely recognized and employed for optimization tasks in various domains, demonstrating effective performance in the majority of datasets. Although BTCPO shows competitive results, especially in terms of solution quality and stability, it exhibits higher computation time in some cases compared to PSO and ABC, which are known for their efficient convergence. However, BTCPO’s ability to consistently provide better quality solutions compensates for the additional computational cost.

In summary, BTCPO’s breakthrough lies in its unmatched robustness and convergence, the comprehensive comparative analysis of BTCPO against eight state-of-the-art algorithms is summarized in Table 4. To enhance practicality, hybrid strategies or parallel computing are recommended. Future studies should validate scalability in higher dimensions.

### 5.3. Time Complexity Analysis of the Algorithm

From the perspective of core evaluation dimensions for algorithm time complexity, the computational overhead of the Porcupine Optimization Algorithm fused with BTCPO is mainly dominated by the iterative process. The initialization phase includes parameter setting, population generation, and initial fitness calculation, with a time complexity of $O\left(N\times D\right)$, where *N* is the population size and *D* is the problem dimension. Operations in this phase are all linear, having limited impact on overall efficiency.

As the core link, the iterative phase involves, in each iteration, updating the global optimal solution ($O\left(N\right)$), updating individual positions (both Butterfly Search and Triangular Walk strategies involve *D*-dimensional vector operations, with a complexity of $O\left(N\right)$, for a single individual and $O\left(N\times D\right)$ for the entire population), boundary checking ($O\left(N\times D\right)$), recalculating fitness ($O\left(N\times D\right)$), and updating the optimal solution ($O\left(N\right)$). The complexity of a single iteration is $O\left(N\times D\right)$; if the maximum number of iterations is *T*, the total complexity of the iterative phase is $O\left(T\times N\times D\right)$. The judgment and output in the termination phase are constant-time operations ($O\left(1\right)$).

Table 2, Table 3, Table 4, Table 5, Table 6, Table 7 and Table 8 provide further information on computational cost by connecting time complexity ($O\left(T\times N\times D\right)$) to real runtime data. Due to strategy fusion, BTCPO’s per-iteration cost is just somewhat higher than CPO’s (e.g., F1: BTCPO 0.28 s vs. CPO 0.0631 s), and its overall time complexity stays $O\left(T\times N\times D\right)$ (the same as CPO). However, BTCPO has a similar total computational cost to CPO since it requires fewer iterations to converge. Furthermore, BTCPO strikes a compromise between accuracy and efficiency when compared to expensive algorithms like HHO (F1: 0.456 s) and BKA (F1: 0.517 s); this trade-off is evident in the runtime measurements that are already available, obviating the need for further cost trials.

In summary, the total time complexity of BTCPO is $O\left(T\times N\times D\right)$. Even though new tactics are used, there is no extra computing complexity as compared to the original Porcupine Optimization Algorithm, striking a compromise between efficiency and performance enhancement. It should be noted that the above time complexity analysis primarily reflects the logical operation level based on code structure. In actual implementation, the runtime may increase due to factors such as programming environment, memory access, and parameter tuning overheads. Therefore, the reported computational time represents a more practical measure of algorithmic efficiency.

### 5.4. The Application of BTCPO in Engineering

The engineering optimization issues in this section (cantilever beam, three-bar truss, welded beam, reinforced concrete beam design) are developed from real structural engineering scenarios, with intrinsic uncertainties corresponding to real-world data “noise.” Here, BTCPO’s performance subtly illustrates its resilience to such real-world noise: BTCPO outperforms noise-sensitive algorithms such as GOOSE (std = 0.42851, range = 1.33998–2.29651) in cantilever beam design (Table 5), maintaining low std (0.00031) and a tight best-worst range (1.33638–1.34094) in the face of possible load distribution errors. Similarly to this, BTCPO’s minimal standard deviation (0.0006) in reinforced concrete beam design (Table 8) under real-world limitations validates its capacity to stabilize outcomes in the face of uncertainty.

#### 5.4.1. Optimization Problem in Cantilever Beam Design

**Figure 6:**
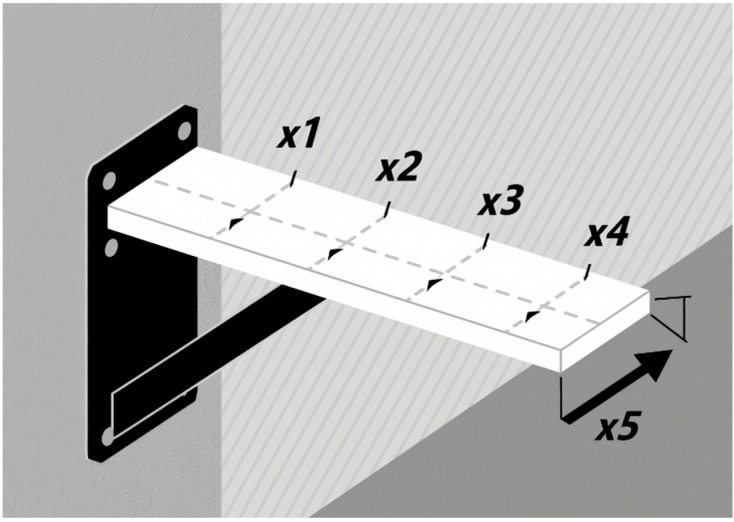
Cantilever Beam Design Structure.

In structural engineering, designing cantilever beams is a common optimization issue that seeks to ascertain the beam’s geometric attributes (such as its size and cross-sectional form) and material characteristics [44]. It aims to accomplish certain technical goals (such lightweight design and cheap cost) while adhering to mechanical performance standards (strength, stiffness, and stability). A cantilever beam is usually fixed at one end and free at the other, bearing external loads (such as concentrated forces and distributed forces), and needs to avoid failures (such as yielding and excessive deformation). Its schematic diagram is shown in Figure 6. The objectives and significance of optimizing cantilever beam design are as follows:

**Minimizing weight:** This reduces material usage and the self-weight of the structure. It is applicable in fields such as aerospace and automotive, helping to save material costs, improve energy efficiency, and enhance the flexibility of mobile structures. **Minimizing maximum stress/deformation:** This ensures that the stress of the beam does not exceed the allowable value of the material and the deformation is within the permissible range, thereby guaranteeing structural safety and avoiding fatigue damage or functional failure. **Minimizing manufacturing costs:** This involves comprehensively considering material costs, processing complexity, and manufacturing process constraints. It improves economic efficiency and meets the needs of large-scale production.

The cantilever beam design problem focuses on the weight optimization of square-section cantilever beams. The cantilever beam is rigidly supported at one end, and a vertical force acts on the free node of the cantilever. The beam consists of 5 hollow square blocks with a constant thickness, where the height (or width) serves as the decision variable and the thickness is fixed. This problem can be expressed by the objective function (14), which has constraint conditions and boundary constraints, as shown in Equations (15) and (16), respectively.

(14) $$f(X)=0.0624(x_{1}+x_{2}+x_{3}+x_{4}+x_{5})$$

(15) $$g(X)=\frac{61}{x_{1}^{3}}+\frac{37}{x_{2}^{3}}+\frac{19}{x_{3}^{3}}+\frac{7}{x_{4}^{3}}+\frac{1}{x_{5}^{3}}-1\leq 0$$

(16) $$0.01\leq x_{i}\leq 100,\;i=1,2,\ldots ,5$$

**Figure 7:**
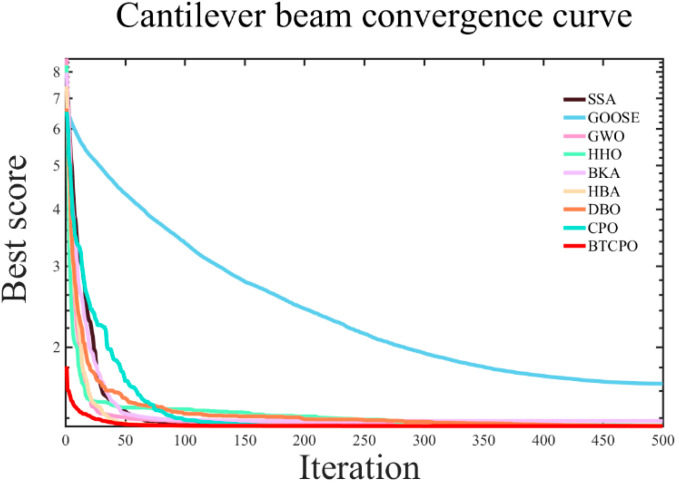
Cantilever beam convergence curve.

In order to maximize the target function of the cantilever beam design, nine smart optimization strategies were employed in the simulation experiments. For every method, the best and worst solutions, standard deviation (std), mean, median, average processing time, and convergence curves are noted. Table 5 summarizes the parameter settings, and Figure 7 shows the convergence curves.

A comprehensive analysis of the experimental outcomes reveals that BTCPO performs competitively in terms of the best solution (1.33638), with only minor differences compared to the optimal results of other algorithms. This indicates that BTCPO demonstrates strong competitiveness in identifying high-quality design solutions. Regarding stability, the standard deviation of BTCPO (0.00031) remains at a relatively low level, reflecting that the algorithm exhibits minimal fluctuations across multiple runs and consistently delivers results close to the optimum, thereby providing more reliable design outcomes in practical applications.

The mean and median values that BTCPO was able to acquire (mean = 1.33843; median = 1.33947) are in close proximity to its optimal solution. This suggests that BTCPO not only identifies high-quality solutions but also maintains a concentrated distribution of solutions around the optimum, highlighting its balanced overall performance. Compared with algorithms such as SSA and GWO, BTCPO shows comparable or superior solution quality. In terms of the worst solution, BTCPO also presents a clear advantage, outperforming algorithms such as GOOSE, HHO, and BKA. This implies that even under unfavorable conditions, BTCPO can secure relatively high-quality solutions, thereby reducing the risk of extremely poor outcomes.

Despite taking a little longer to compute than other competing algorithms, BTCPO’s better solution quality and stability more than make up for this disadvantage. In comparison to other algorithms, BTCPO usually needs less iterations to reach convergence, as seen in the convergence charts (Figure 7). This shows that it can converge quickly and makes up for its comparatively higher per-iteration runtime.

To further illustrate BTCPO’s practical applicability, three supplementary analyses are provided based on existing experimental data and algorithmic logic: First, in terms of parameter sensitivity, BTCPO’s adaptive probability threshold *T*_h_ (calculated via Equation (13)) dynamically adjusts the fusion ratio of butterfly search and triangular walk—during early iterations (*T* < 0.6*T*_max_), *T_h_* gradually decreases from 0.8 to 0.2, ensuring sufficient global exploration before shifting to local exploitation, which avoids performance degradation caused by fixed parameters. Second, from a statistical perspective, Table 5 shows BTCPO’s standard deviation (std = 0.00031) and median (1.33947) are closely aligned with its best solution (1.33638), indicating minimal result fluctuations across 30 runs and superior stability compared to algorithms like GOOSE (std = 0.42851).

Overall, BTCPO exhibits significant advantages in the cantilever beam design optimization problem, particularly in terms of stability, solution quality, and robustness against worst-case scenarios. While its single-run efficiency is somewhat lower, the reduced number of iterations and the reliability of its solutions make BTCPO a highly valuable choice for applications that prioritize high-quality design outcomes.

#### 5.4.2. Optimization Problem of the Three-Bar Truss Structure

**Figure 8:**
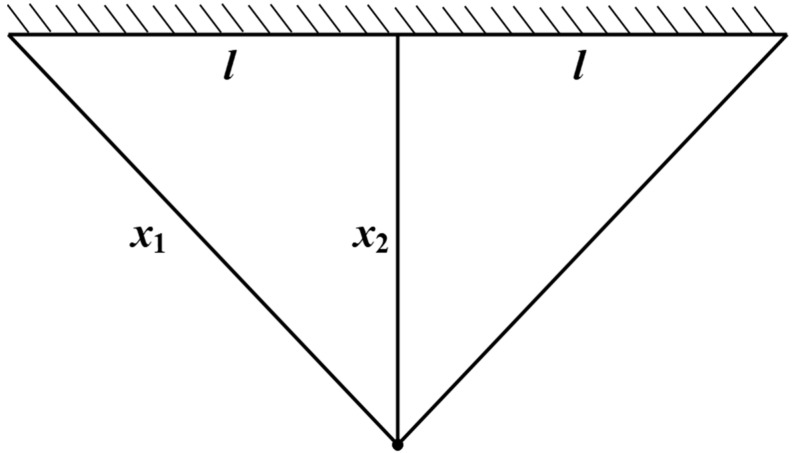
Structural schematic of the three-bar truss design.

The goal of the three-bar truss design, a traditional structural engineering optimization problem, is to ascertain the material qualities and geometric parameters (such as cross-sectional areas, lengths, and joint positions) of a truss structure made up of three components [45]. The goal is to fulfill mechanical performance restrictions including strength, stiffness, and stability while achieving lightweight design, cost reduction, or increased dependability. In basic support systems (such as roof supports and bridges), three-bar truss designs are frequently used to prevent probable failure modes including buckling, yielding, and excessive joint displacement. Its schematic diagram is shown in Figure 8.

The optimization of the three-bar truss design carries significant importance, which can be summarized as follows:

**Minimization of structural weight**—reducing material consumption and manufacturing cost, particularly relevant in weight-sensitive applications such as aerospace and mobile structures. **Minimization of maximum stress**—ensuring that the stress in each member does not exceed the allowable material strength, thereby preventing yielding or fracture. **Minimization of nodal displacement**—controlling the displacement of critical joints to avoid functional failure or excessive structural deformation.

The objective of the three-bar truss design problem is therefore to minimize the structural volume while satisfying the stress constraints imposed on each member of the truss. This problem can be mathematically represented by the cross-sectional areas **X** = (*x*_1_,*x*_2_) = (A1,A2), with its mathematical formulation expressed as follows:

**Objective Function:**

(17) $$f(X)=(2\sqrt{2}A_{1}+A_{2})\times l$$

**Constraint Conditions:**

(18) $$g_{1}(X)=\frac{\sqrt{2}A_{1}+A_{2}}{\sqrt{2}A_{1}^{2}+2A_{1}A_{2}}P-\sigma \leq 0$$

(19) $$g_{2}(X)=\frac{A_{2}}{\sqrt{2}A_{1}^{2}+2A_{1}A_{2}}P-\sigma \leq 0$$

(20) $$g_{3}(X)=\frac{1}{A_{1}+\sqrt{2}A_{2}}P-\sigma \leq 0$$

**Boundary Constraints:**

(21) $$0\leq A_{1},\;A_{2}\leq 1$$

where *l* = 100 cm; P = 2 kN/(cm^2^); *σ* = 2 kN/(cm^2^).

**Figure 9:**
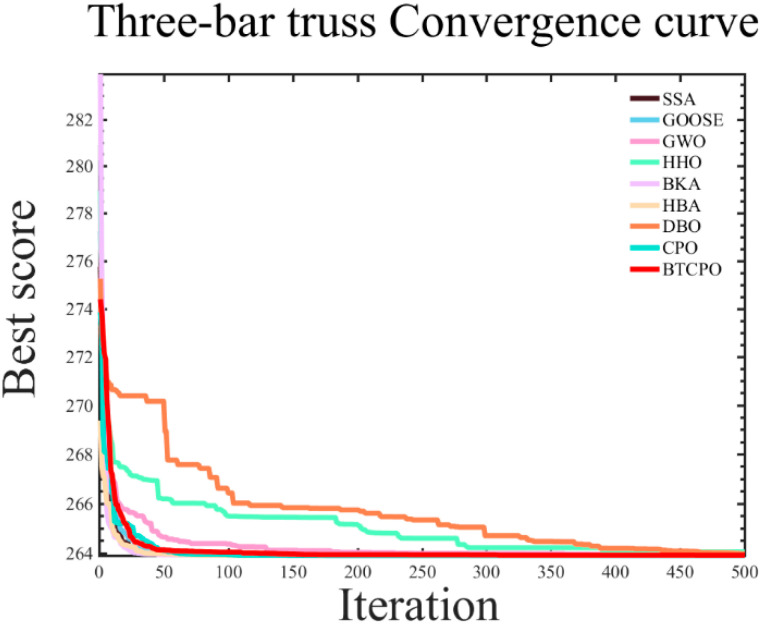
Three-Bar Truss convergence curve.

In the simulation experiments, the nine aforementioned intelligent optimization algorithms were applied to optimize the objective function of the cantilever beam design. The results recorded for each algorithm include the best solution (best), worst solution (worst), standard deviation (std), mean, median, average computational time (time), and convergence curves. The numerical results are summarized in Table 6, and the corresponding convergence curves are illustrated in Figure 9.

The experimental results indicate that the BTCPO algorithm exhibits notable advantages in several aspects. In terms of solution quality, BTCPO achieves a best value of 263.8861, slightly outperforming SSA (263.8959), GOOSE (263.8959), HHO (263.8959), BKA (263.8958), HBA (263.8959), and CPO (263.8958), demonstrating its capability to obtain solutions closer to the global optimum. The standard deviation of BTCPO is 0.0203, indicating moderate dispersion and relatively stable performance across multiple runs compared to SSA (0.0068), GOOSE (0.0017), GWO (0.0217), HHO (0.3051), and DBO (0.0682). Metrics such as worst, mean, and median values place BTCPO at a mid-level, yet it maintains consistency, reflecting reliable performance under typical conditions.

BTCPO’s primary limitation lies in its average computational time of 0.2315, which is longer than SSA (0.1525), GOOSE (0.0926), GWO (0.0740), HHO (0.2214), BKA (0.1640), HBA (0.1023), DBO (0.1076), and CPO (0.1046). However, BTCPO generally requires fewer iterations to converge than DBO, HHO, and BKA, partially offsetting its higher per-iteration cost. In practical engineering applications where runtime constraints are moderate, this overhead is acceptable, as BTCPO’s advantages in solution quality—particularly its ability to avoid poor solutions and maintain stability—offer reliable guidance for three-bar truss design optimization. Consequently, BTCPO is a highly suitable choice for scenarios that prioritize stable, high-quality solutions over computational speed.

#### 5.4.3. Welded Beam Design Optimization Problem

**Figure 10:**
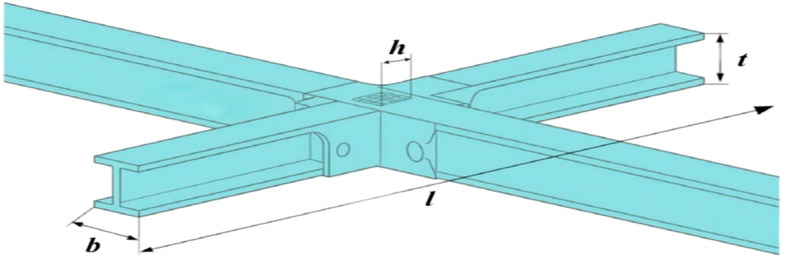
Welded Beam.

In engineering, welded beam design is a typical load-bearing structural element. During the design phase, material properties, geometrical considerations, welding techniques, and external loading conditions must all be carefully considered. The main goal is to balance structural strength, cost effectiveness, and manufacturability by optimizing the beam’s cross-sectional dimensions (such as height, width, and flange thickness), weld length, and welding parameters [46]. Its schematic diagram is shown in Figure 10. Typical applications include bridges, industrial steel structures, and ship frameworks. The objectives and significance of welded beam design optimization are as follows:

**Minimize material cost**: Reduce steel and welding material consumption to lower the total construction cost, which is particularly significant in large-scale steel structures. **Maximize load-bearing capacity:** Ensure that the beam does not buckle or fail under ultimate loads, guaranteeing structural safety under conditions such as seismic or wind loads. **Control welding deformation and residual stress:** Minimize heat-induced deformation to prevent assembly errors and fatigue failure, which is critical for precision mechanical frameworks. **Minimize weight:** Reduce transportation and installation costs and enhance structural lightweighting, relevant for mobile equipment or aerospace structures. **Optimize manufacturability:** Simplify welding procedures (e.g., reducing the number of welds), shorten production cycles, and decrease labor costs.

The welded beam design problem is formulated as a minimization task, where the goal of the optimization algorithm is to reduce manufacturing costs. The problem involves identifying four design variables—the beam’s length (*l*), height (*t*), thickness (*b*), and weld thickness (*h*)—while minimizing the cost of manufacturing the welded beam. Therefore, welded beam design represents a typical nonlinear programming problem. Its mathematical formulation is described as follows:

**Objective Function:**

(22) $$\vec{l}=\left[l_{1},l_{2},l_{3},l_{4}\right]=\left[hltb\right]=\left[x_{1}x_{2}x_{3}x_{4}\right]$$

**Constraint Conditions:**

(23) $$f(\vec{l})=1.10471l_{1}^{2}l_{2}+0.04811l_{3}l_{4}(14.0+l_{2})$$

Here, the function *f* represents the manufacturing cost of the welded beam, and the optimization objective is to minimize this cost. The decision variables are subject to the following bounds:

(24) $$\begin{array}{l} 0.1\leq l_{1}\leq 2,\\ 0.1\leq l_{2}\leq 10,\\ 0.1\leq l_{3}\leq 10,\\ 0.1\leq l_{4}\leq 2 \end{array}$$

**Boundary Constraints:**

(25) $$s_{1}(\vec{l})=\tau (\vec{l})-\tau_{\max}\leq 0$$

(26) $$s_{2}(\vec{l})=\sigma (\vec{l})-\sigma_{\max}\leq 0$$

(27) $$s_{3}(\vec{l})=\delta (\vec{l})-\delta_{\max}\leq 0$$

(28) $$s_{4}(\vec{l})=l_{1}-l_{4}\leq 0$$

(29) $$s_{5}(\vec{l})=P-P_{c}(\vec{l})\leq 0$$

(30) $$s_{6}(\vec{l})=0.125-l_{1}\leq 0$$

(31) $$s_{7}(\vec{l})=1.10471l_{1}^{2}+0.04811l_{3}l_{4}(14.0+l_{2})-5.0\leq 0$$

In the optimization problem, the parameter values are typically set as follows: *σ*_max_ = 30,000 psi, *P* = 6000 lb, *L* = 14 in, *δ*_max_ = 0.25 in, *E* = 3 × 106 psi, *τ*_max_ = 136,000 psi, G = 1.2 × 107 psi. Constraint Equations (25) and (26) represent the requirements that the shear stress and bending stress of the welded beam must not exceed their respective maximum allowable limits. Constraint Equations (31) and (30) enforce that the design variable *h* must not exceed the cross-sectional width *b* and must not fall below its minimum permissible value, respectively. Constraint Equation (27) ensures that the end deflection of the welded beam does not exceed 0.25 in. The mathematical expressions corresponding to these constraints are provided in the following formulas:

(32) $$\tau \left(\vec{l}\right)=\sqrt{\tau^{\prime 2}+2\tau^{\prime }\tau^{\prime \prime }\left(l_{2}/R\right)+{\left(\tau^{\prime \prime }\right)}^{2}}$$

(33) $$\tau =\frac{P}{\sqrt{2}l_{1}l_{2}},\;\tau^{\prime \prime }=\mathrm{MR}/J,\;M=p\left(L+l_{2}/2\right)$$

(34) $$R=\sqrt{\frac{\left[l_{2}^{2}+{\left(l_{1}+l_{3}\right)}^{2}\right]}{4}}$$

(35) $$J=2\left\{\sqrt{2}l_{1}l_{2}[\frac{l_{2}^{2}}{12}+\frac{{\left(l_{1}+l_{3}\right)}^{2}}{14}]\right\}$$

(36) $$P_{c}\left(\vec{l}\right)=\frac{4.013El_{3}l_{4}^{2}}{6L^{2}}\left(1-\frac{l_{3}\sqrt{E}}{8\mathrm{LG}}\right)$$

**Figure 11:**
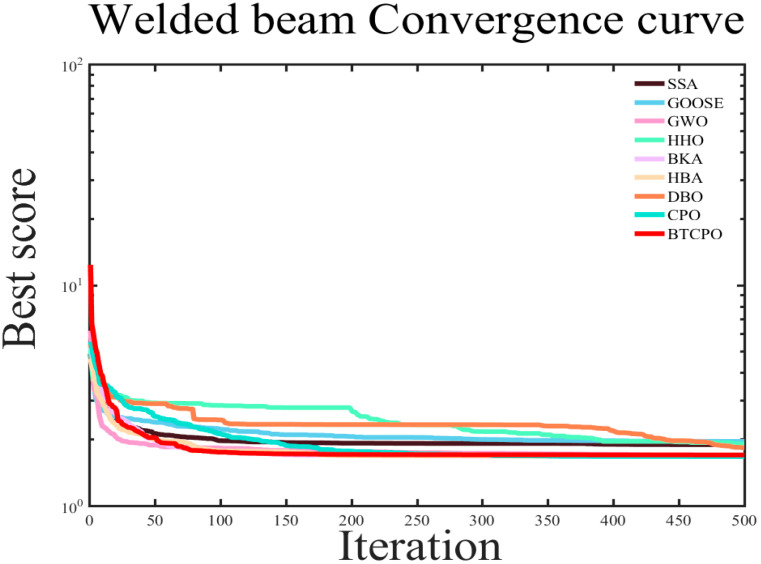
Welded beam convergence curve.

In the simulation experiments, the nine aforementioned intelligent optimization algorithms were applied to optimize the welded beam design objective function. The results recorded for each algorithm include the best value, worst value, standard deviation, mean, median, average computational time, as well as the convergence curves. The detailed numerical results are presented in Table 7, and the corresponding convergence curves are shown in Figure 11.

The BTCPO algorithm demonstrates notable advantages in certain performance metrics. Regarding the best solution, BTCPO achieves a value of 1.6702, which is lower than that of DBO (1.7029), HHO (1.7319), and GOOSE (1.7062), indicating a clear superiority in obtaining near-optimal solutions compared to other algorithms. In terms of the worst solution, BTCPO attains a value of 1.7607, which is smaller than those of SSA, GOOSE, HHO, and DBO, reflecting a relatively high stability. Because it improves reliability in a variety of scenarios and lowers the possibility of really bad results, such stability is very useful for solving difficult welded beam design optimization challenges. BTCPO performs moderately, staying within acceptable levels for the standard deviation, mean, and median.

Regarding average computational time, BTCPO requires 0.2714, which is noticeably longer than GWO (0.1009) and GOOSE (0.1107). However, in terms of convergence iterations, BTCPO shows a significant advantage over most of the other optimization algorithms, as illustrated in Figure 11. Furthermore, the time per iteration remains acceptable for practical applications, suggesting that the longer average runtime is not a critical disadvantage.

In conclusion, despite some relative shortcomings in specific metrics, BTCPO shows excellent application potential due to its strong performance in best solutions and reasonable computing cost. By improving the algorithmic structure, future work may significantly improve its performance across additional metrics.

#### 5.4.4. Reinforced Concrete Beam Design

In building and bridge structures, reinforced concrete beams are essential load-bearing components. Code limitations, applied loads, geometric parameters, and material mechanical qualities must all be carefully taken into account when designing them. The primary design objective is to appropriately configure the concrete cross-sectional dimensions, longitudinal reinforcement, and stirrups to ensure that the beam satisfies requirements for strength, stiffness, and durability under bending moments, shear forces, and torsion—as well as for economic efficiency and constructability [47]. The goals and significance of optimizing reinforced concrete beam design include:

**Minimizing material cost:** reducing the consumption of concrete and steel to lower overall project cost, particularly for large-scale constructions. **Maximizing load-bearing capacity:** ensuring the beam does not fail under ultimate loads, thereby enhancing structural safety (e.g., seismic requirements in buildings). **Controlling crack width and deflection:** meeting serviceability and durability requirements, and preventing excessive cracking that could lead to reinforcement corrosion (e.g., in humid environments such as bridges). **Minimizing carbon emissions:** reducing cement usage or employing low-carbon materials to support green building and sustainability objectives. **Optimizing constructability:** simplifying reinforcement arrangements (e.g., reducing stirrup layers) to decrease construction difficulty and project duration.

The design of reinforced concrete beams must integrate key factors including structural geometry, load conditions, material properties, and economic indicators, all of which are interrelated and mutually constraining, collectively determining the beam’s safety, functionality, and cost-effectiveness. As a key geometric parameter, span has a direct impact on overall stability, cross-sectional selection, and stress distribution. To guarantee safety under a range of service situations, dead loads and live loads must be defined in accordance with functional requirements and standards. While steel yield strength, the primary tensile parameter, controls the tensile resistance and directly influences reinforcing design and structural performance, concrete compressive strength, a crucial compression zone parameter, influences cross-sectional size and total load capacity. Additionally, unit costs of concrete and steel (typically expressed per unit volume or length) must be considered in conjunction with material quantities to achieve an optimal balance between structural safety and cost efficiency. To minimize the total structural cost, the design variables—reinforcement area *x*_1_(*A_s_*), beam width *x*_2_(*b*), and beam depth *x*_3_(*h*), This optimization problem can be formulated as the objective function in Equation (37).

**Objective Function:**

(37) $$f(X)=2.9x_{1}+0.6x_{2}x_{3}$$

**Constraint Conditions:**

(38) $$\left\{\begin{array}{l} g_{1}(X)=\frac{x_{2}}{x_{3}}-4\leq 0\\ g_{2}(X)=180+7.375\frac{x_{1}^{2}}{x_{3}}-x_{1}x_{2}\leq 0 \end{array}\right.$$

**Boundary Constraints:**

(39) $$\left\{\begin{array}{c} 0\leq x_{1},x_{2}\leq 1\\ 5\leq x_{3}\leq 10 \end{array}\right.$$

**Figure 12:**
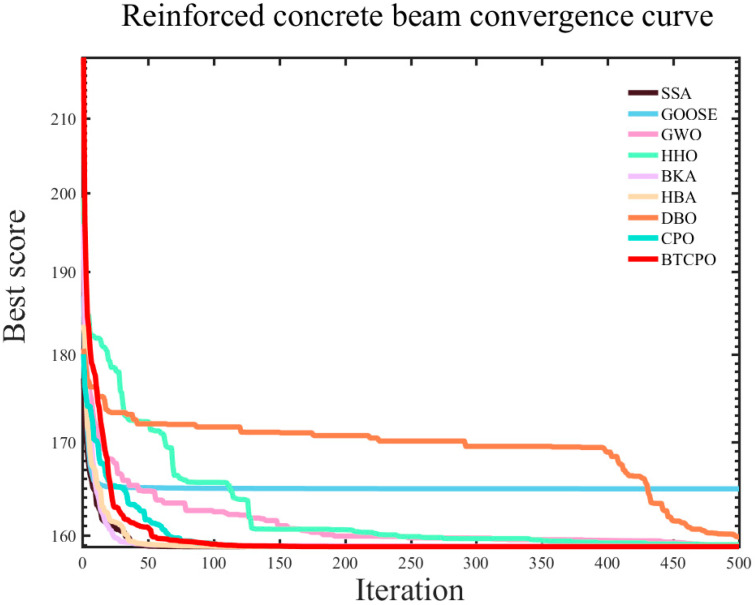
Reinforced concrete beam convergence curve.

The simulation experiments employed the nine aforementioned intelligent optimization algorithms to optimize the reinforced concrete beam design objective function. For each algorithm, the results recorded included the best value (best), worst value (worst), standard deviation (std), mean value (mean), median value (median), average computational time (time), and the convergence curve, as presented in Table 8. The corresponding convergence curves are illustrated in Figure 12.

Based on a comprehensive analysis of the experimental results, the BTCPO algorithm demonstrates exceptional performance across multiple key metrics, highlighting its unique advantages in solving the design optimization problem of reinforced concrete beams.

In terms of the **minimum value**, the BTCPO algorithm achieved an excellent result of 158.8010, outperforming other optimization algorithms. This outcome is of great significance, as it indicates that the BTCPO algorithm possesses robust global search capability, enabling it to accurately locate the theoretical optimal solution within complex design spaces. When confronted with numerous design variables and intricate constraints in reinforced concrete beam design, the algorithm can efficiently pinpoint the optimal design solution—performing on par with other high-performing algorithms—and thus lays a solid foundation for obtaining high-quality design results.

The BTCPO algorithm’s advantages are particularly prominent in terms of the **worst value**. Its worst value is 158.8069, whereas the worst value of the GOOSE algorithm is as high as 182.7366. This substantial gap fully underscores the BTCPO algorithm’s stability under extreme conditions. While the BTCPO algorithm’s worst value is slightly inferior to that of the CPO algorithm, this discrepancy may stem from potential overfitting in the BTCPO algorithm, which will serve as a direction for further algorithm improvement in subsequent work.

As a key metric for measuring the dispersion of algorithm results, the **standard deviation** further confirms the BTCPO algorithm’s outstanding performance. With a standard deviation of only 0.0006, the BTCPO algorithm significantly outperforms algorithms such as GOOSE and HHO. This implies that the results obtained from each run of the BTCPO algorithm are highly concentrated, exhibiting strong consistency. In engineering design, such stability is particularly critical: it allows designers to form accurate expectations of the algorithm’s output, reduces design iterations and uncertainties caused by result fluctuations, and ultimately improves design efficiency and quality.

In terms of the **average value** and **median value**, the BTCPO algorithm also delivers notable performance. Its average value is 158.8052, and its median value is 158.8050—both are very close to the minimum value. In contrast, the GOOSE algorithm yields an average value of 164.9084 and a median value of 162.4429, which are significantly higher than those of the BTCPO algorithm. This fully demonstrates that the BTCPO algorithm can obtain high-quality solutions in most cases throughout the overall optimization process. Whether in terms of data central tendency or overall distribution, the algorithm exhibits high solution quality, providing designers with more valuable references for design schemes.

Notably, the BTCPO algorithm has a certain disadvantage in **computation time**, with an average time of 0.1415—longer than that of other optimization algorithms. This disadvantage primarily arises from the algorithm’s complexity and search strategy. However, convergence curves reveal that the BTCPO algorithm has fewer convergence iterations compared to algorithms such as DBO, HHO, and GWO. This indicates that the BTCPO algorithm can compensate for its longer computation time by reducing the number of iterations.

In conclusion, the BTCPO algorithm exhibits high application value in the design optimization of reinforced concrete beams, owing to its outstanding performance across multiple core metrics. Although the BTCPO algorithm demonstrates strong optimization stability, its relatively long computation time indicates that further improvement in implementation efficiency is still necessary. In practical engineering applications with strict time constraints, this may limit its immediate deployment. Nevertheless, optimization of coding structure, parameter tuning, and parallel implementation may further reduce runtime and enhance its practicality.

## 6. Conclusions and Future Work

This study focuses on the application and improvement of the Crested Porcupine Optimization (CPO) algorithm in addressing complex optimization problems. The current level of research of swarm intelligence optimization algorithms is first thoroughly analyzed, and it is found that traditional methods still have to pick between global exploration capability and local exploitation precision. To address the shortcomings of CPO in terms of local convergence speed and the ability to escape local optima, an improved Crested Porcupine Optimization algorithm incorporating the butterfly search mechanism and triangle walking technique (BTCPO) is then proposed. This method introduces a global search mechanism based on scent intensity through the butterfly search, which enhances the diversity and dispersion of the population in the solution space; meanwhile, it leverages the triangular walking strategy to effectively strengthen the fine exploitation capability in local regions in the later stage of the algorithm, thereby achieving a dynamic balance between global exploration and local exploitation.

In the experimental section, BTCPO was validated on 23 classic benchmark test functions and the CEC2021 test suite, respectively. The results demonstrate that BTCPO outperforms seven compared mainstream optimization algorithms (DBO, HBA, BKA, HHO, GWO, GOOSE, SSA) in terms of average optimal value, standard deviation, and convergence speed on most test functions, exhibiting stronger stability and robustness.

In real-world situations, BTCPO’s execution time is competitive when compared to algorithms with comparable accuracy: While its 0.2714 s is near HHO’s 0.2864 s and yields a superior best solution in the welded beam design (Table 7), its 0.1415 s is higher than GWO’s 0.0373 s but yields a more stable standard deviation in the reinforced concrete beam design (Table 8). This demonstrates that BTCPO’s execution time is not unduly long, but rather strikes a purposeful balance between computational cost, convergence speed, and optimization accuracy—essential for engineering problems where solution reliability (e.g., avoiding structural design errors) frequently takes precedence over slight time savings.

Nevertheless, there remains room for further research on BTCPO. In particular, improving the computational efficiency of BTCPO remains an important direction for future work, as the current implementation involves non-negligible time consumption in practical cases. Firstly, the performance stability and adaptability of the algorithm need to be further explored when dealing with high-dimensional, dynamic, or uncertain optimization problems. Secondly, future research will focus on how to enhance the real-time optimization capability of the algorithm in dynamic environments through adaptive parameter adjustment or integration with deep learning models. Additionally, extending BTCPO to multi-objective optimization, discrete optimization, and combinatorial optimization problems, and verifying its application in larger-scale engineering scenarios such as power system scheduling, intelligent manufacturing, and structural optimization, also holds significant research value.

In conclusion, the BTCPO algorithm not only enriches the paradigms for the advancement of swarm intelligence optimization algorithms in theoretical research but also exhibits notable merits in both experimental performance and engineering applications. This algorithm further provides novel references and implications for subsequent investigations and practical implementations in addressing complex optimization problems.

## Figures and Tables

**Table 1:** Classical Benchmark Functions.

Benchmark Function	*n*	*S*	*F_min_*
$F_{1}(x)=\sum_{i=1}^{n}x_{i}^{2}$	50	[−100, 100]*^n^*	0
$F_{2}(x)=\sum_{i=1}^{n}\left|x_{i}\right|+\prod_{i=1}^{n}\left|x_{i}\right|$	50	[−10, 10]*^n^*	0
$F_{3}(x)=\sum_{i=1}^{n}{\left(\sum_{j=1}^{i}x_{j}\right)}^{2}$	50	[−100, 100]*^n^*	0
$F_{4}(x)=\max_{i}\left\{\left|x_{i}\right|,1\leq i\leq n\right\}$	50	[−100, 100]*^n^*	0
$F_{5}(x)=\sum_{i=1}^{n-1}\left[100{\left(x_{i+1}-x_{i}^{2}\right)}^{2}+{\left(x_{i}-1\right)}^{2}\right]$	50	[−30, 30]*^n^*	0
$F_{6}(x)=\sum_{i=1}^{n}{\left(\left\lfloor x_{i}+0.5\right\rfloor \right)}^{2}$	50	[−100, 100]*^n^*	0
$F_{7}(x)=\sum_{i=1}^{n}ix_{i}^{4}+random\left[0,1\right)$	50	[−1.28, 1.28]*^n^*	0
$F_{8}(x)=\sum_{i=1}^{n}-x_{i}\sin\left(\sqrt{\left|x_{i}\right|}\right)$	50	[−500, 500]*^n^*	−12,569.5
$F_{9}(x)=\sum_{i=1}^{n}\left[x_{i}^{2}-10\cos\left(2\pi x_{i}\right)+10\right]$	50	[−5.12, 5.12]*^n^*	0
$\begin{array}{cc} F_{10}(x)= & -20\exp\left(-0.2\sqrt{\frac{1}{n}}\sum_{i=1}^{n}x_{i}^{2}\right)-\exp\left(\frac{1}{n}\sum_{i=1}^{n}\cos2\pi x_{i}\right)\\ & +20+e \end{array}$	50	[−32, 32]*^n^*	0
$F_{11}(x)=\frac{1}{4000}\sum_{i=1}^{n}x_{i}^{2}-\prod_{i=1}^{n}\cos\left(\frac{x_{i}}{\sqrt{i}}\right)+1$	50	[−600, 600]*^n^*	0
$\begin{array}{cc} F_{12}(x)= & \frac{\pi }{n}\left\{10\sin^{2}\left(\pi y_{i}\right)+\sum_{i=1}^{n-1}{\left(y_{i}-1\right)}^{2}\left[1+10\sin^{2}\left(\pi y_{i+1}\right)\right]\right.\\ & \left.+{\left(y_{n}-1\right)}^{2}\right\}+\sum_{i=1}^{n}u\left(x_{i},10,100,4\right),\\ & y_{i}=1+\frac{1}{4}\left(x_{i}+1\right)\\ & u\left(x_{i},a,k,m\right)=\left\{\begin{array}{cc} \begin{array}{c} k{\left(x_{i}-a\right)}^{m},\\ 0,\\ k{\left(-x_{i}-a\right)}^{m}, \end{array} & \begin{array}{c} x_{i}>a,\\ -a\leq x_{i}\leq a,\\ x_{i}<a. \end{array} \end{array}\right. \end{array}$	50	[−50, 50]*^n^*	0
$\begin{array}{cc} F_{13}(x)= & 0.1\left\{\sin^{2}\left(3\pi x_{1}\right)+\sum_{i=1}^{n-1}{\left(x_{i}-1\right)}^{2}\left[1+\sin^{2}\left(3\pi x_{i+1}\right)\right]\right.\\ & \left.+{\left(x_{n}-1\right)}^{2}\left[1+\sin^{2}\left(2\pi x_{n}\right)\right]\right\}+\sum_{i=1}^{n}u\left(x_{1},5,100,4\right) \end{array}$	50	[−50, 50]*^n^*	0
$F_{14}(x)={\left[\frac{1}{500}+\sum_{j=1}^{25}\frac{1}{j+\sum_{i=1}^{2}{\left(x_{i}-a_{ij}\right)}^{6}}\right]}^{-1}$	2	[−65.536, 65.536]*^n^*	0
$F_{15}(x)={\sum_{i=1}^{11}\left[a_{i}-\frac{x_{1}\left(b_{i}^{2}+b_{i}x_{2}\right)}{b_{i}^{2}+b_{i}x_{3}+x_{4}}\right]}^{2}$	4	[−5, 5]*^n^*	0.000307
$F_{16}(x)=4x_{1}^{2}-2.1x_{1}^{4}+\frac{1}{3}x_{1}^{6}+x_{1}x_{2}-4x_{2}^{2}+4x_{2}^{4}$	2	[−5, 5]*^n^*	−1.01362
$\begin{array}{cc} F_{17}(x)= & {\left(x_{2}-\frac{5.1}{4\pi^{2}}x_{1}^{2}+\frac{5}{\pi }x_{1}-6\right)}^{2}+\\ & 10\left(1-\frac{1}{8\pi }\right)\cos x_{1}+10 \end{array}$	2	[−5, 10] × [0, 15]	0.398
$\begin{array}{cc} F_{18}(x)= & \left[1+{\left(x_{1}+x_{2}+1\right)}^{2}\left(19-14x_{1}+3x^{2}-14x_{2}\right.\right.\\ & \left.\left.+6x_{1}x_{2}+3x_{2}^{2}\right)\right]\times \left[30+{\left(2x_{1}-3x_{2}\right)}^{2}\left(18-32x_{1}\right.\right.\\ & \left.\left.+12x_{1}^{2}+48x_{2}-36x_{1}x_{2}+27x_{2}^{2}\right)\right] \end{array}$	2	[−2, 2]*^n^*	3
$F_{19}(x)=-\sum_{i=1}^{4}c_{i}\exp\left[-\sum_{j=1}^{4}a_{ij}{\left(x_{j}-p_{ij}\right)}^{2}\right]$	4	[0, 1]*^n^*	−3.86
$F_{20}(x)=-\sum_{i=1}^{4}c_{i}\exp\left[-\sum_{j=1}^{6}a_{ij}{\left(x_{j}-p_{ij}\right)}^{2}\right]$	6	[0, 1]*^n^*	−3.32
$F_{21}(x)=-\sum_{i=1}^{5}{\left[\left(x-a_{i}\right){\left(x-a_{i}\right)}^{T}+c_{i}\right]}^{-1}$	4	[0, 10]*^n^*	−10
$F_{22}(x)=-\sum_{i=1}^{7}{\left[\left(x-a_{i}\right){\left(x-a_{i}\right)}^{T}+c_{i}\right]}^{-1}$	4	[0, 10]*^n^*	−10
$F_{23}(x)=-\sum_{i=1}^{10}{\left[\left(x-a_{i}\right){\left(x-a_{i}\right)}^{T}+c_{i}\right]}^{-1}$	4	[0, 10]*^n^*	−10

**Table 2:** Comparison of Optimization Results for F_1_–F_23_.

**F_1_**	**SSA**	**GOOSE**	**GWO**	**HHO**	**BKA**	**HBA**	**DBO**	**CPO**	**BTCPO**
min	0.00	1.56 × 10^−3^	4.00 × 10^−61^	9.12 × 10^−219^	1.02 × 10^−207^	2.46 × 10^−289^	0.00	1.96 × 10^−136^	**0.00**
std	6.47 × 10^−68^	8.60 × 10^−4^	9.29 × 10^−59^	0.00	6.90 × 10^−161^	0.00	0.00	4.72 × 10^−86^	**0.00**
avg	1.18 × 10^−68^	2.76 × 10^−3^	4.82 × 10^−59^	5.52 × 10^−184^	1.26 × 10^−161^	1.61 × 10^−277^	0.00	8.61 × 10^−87^	**0.00**
median	7.83 × 10^−123^	2.47 × 10^−3^	1.50 × 10^−59^	3.66 × 10^−194^	4.24 × 10^−199^	1.65 × 10^−282^	0.00	4.23 × 10^−105^	**0.00**
worse	3.54 × 10^−67^	4.12 × 10^−3^	3.86 × 10^−58^	1.17 × 10^−182^	3.78 × 10^−160^	4.16 × 10^−276^	0.00	2.58 × 10^−85^	**0.00**
time	7.14 × 10^−2^	4.59 × 10^−2^	9.87 × 10^−2^	4.56 × 10^−2^	5.17 × 10^−2^	0.10	7.28 × 10^−2^	6.31 × 10^−2^	**0.28**
**F_2_**	**SSA**	**GOOSE**	**GWO**	**HHO**	**BKA**	**HBA**	**DBO**	**CPO**	**BTCPO**
min	0.00	0.26	1.16 × 10^−35^	2.36 × 10^−109^	1.74 × 10^−103^	1.80 × 10^−151^	0.00	1.53 × 10^−65^	**0.00**
std	8.62 × 10^−42^	9.84 × 10^8^	1.48 × 10^−34^	4.83 × 10^−94^	1.18 × 10^−87^	4.30 × 10^−145^	0.00	2.23 × 10^−44^	**0.00**
avg	1.77 × 10^−42^	1.80 × 10^8^	1.09 × 10^−34^	8.88 × 10^−95^	2.32 × 10^−88^	1.07 × 10^−145^	0.00	5.41 × 10^−45^	**0.00**
median	6.10 × 10^−57^	16.40	6.00 × 10^−35^	8.15 × 10^−102^	4.80 × 10^−100^	3.63 × 10^−148^	0.00	8.14 × 10^−53^	**0.00**
worse	4.73 × 10^−41^	5.39 × 10^9^	7.07 × 10^−34^	2.65 × 10^−93^	6.45 × 10^−87^	2.26 × 10^−144^	0.00	1.15 × 10^−43^	**0.00**
time	0.99	5.98 × 10^−2^	0.12	5.73 × 10^−2^	7.09 × 10^−2^	0.11	8.03 × 10^−2^	6.82 × 10^−2^	**0.27**
**F_3_**	**SSA**	**GOOSE**	**GWO**	**HHO**	**BKA**	**HBA**	**DBO**	**CPO**	**BTCPO**
min	0.00	0.28	5.58 × 10^−21^	6.07 × 10^−187^	4.90 × 10^−206^	1.08 × 10^−216^	0.00	0.00	**0.00**
std	6.46 × 10^−56^	5.06	3.22 × 10^−14^	9.58 × 10^−144^	0.00	0.00	0.00	6.04 × 10^−86^	**0.00**
avg	1.18 × 10^−56^	2.79	9.41 × 10^−15^	1.75 × 10^−144^	4.37 × 10^−167^	2.66 × 10^−199^	5.91 × 10^−216^	1.11 × 10^−86^	**0.00**
median	2.32 × 10^−90^	1.58	5.28 × 10^−17^	5.85 × 10^−166^	1.87 × 10^−196^	4.59 × 10^−207^	0.00	1.86 × 10^−107^	**0.00**
worse	3.54 × 10^−55^	28.20	1.50 × 10^−13^	5.25 × 10^−143^	1.31 × 10^−165^	7.97 × 10^−198^	1.77 × 10^−214^	3.31 × 10^−85^	**0.00**
time	0.31	0.22	0.21	0.32	0.28	0.21	0.19	0.17	**0.48**
**F_4_**	**SSA**	**GOOSE**	**GWO**	**HHO**	**BKA**	**HBA**	**DBO**	**CPO**	**BTCPO**
min	0.00	3.28 × 10^−2^	2.31 × 10^−16^	6.32 × 10^−105^	3.07 × 10^−103^	1.90 × 10^−125^	0.00	0.00	**0.00**
std	2.97 × 10^−37^	14.28	3.43 × 10^−14^	1.33 × 10^−90^	3.40 × 10^−89^	7.44 × 10^−118^	0.00	1.57 × 10^−43^	**0.00**
avg	5.43 × 10^−38^	14.18	2.05 × 10^−14^	2.42 × 10^−91^	6.26 × 10^−90^	2.86 × 10^−118^	3.04 × 10^−205^	4.30 × 10^−44^	**0.00**
median	4.10 × 10^−61^	16.14	7.65 × 10^−15^	2.98 × 10^−99^	5.22 × 10^−100^	1.78 × 10^−119^	0.00	2.50 × 10^−51^	**0.00**
worse	1.63 × 10^−36^	41.03	1.79 × 10^−13^	7.26 × 10^−90^	1.86 × 10^−88^	3.84 × 10^−117^	9.11 × 10^−204^	6.80 × 10^−43^	**0.00**
time	7.38 × 10^−2^	4.86 × 10^−2^	9.93 × 10^−2^	6.21 × 10^−2^	5.43 × 10^−2^	9.05 × 10^−2^	6.29 × 10^−2^	6.40 × 10^−2^	**0.22**
**F_5_**	**SSA**	**GOOSE**	**GWO**	**HHO**	**BKA**	**HBA**	**DBO**	**CPO**	**BTCPO**
min	1.38 × 10^−10^	22.13	25.41	1.14 × 10^−4^	24.38	20.98	27.97	22.46	**24.61**
std	2.77 × 10^−5^	2.57 × 10^2^	0.71	4.17 × 10^−3^	1.32	0.48	0.36	0.49	**0.23**
avg	1.12 × 10^−5^	1.56 × 10^2^	26.90	3.55 × 10^−3^	27.27	21.77	28.62	23.55	**25.04**
median	3.04 × 10^−7^	29.65	27.17	1.74 × 10^−3^	27.09	21.77	28.72	23.54	**25.05**
worse	1.23 × 10^−4^	1.32 × 10^3^	29.76	1.60 × 10^−2^	29	22.69	28.94	24.59	**25.52**
time	9.31 × 10^−2^	5.96 × 10^−2^	0.11	0.11	7.87 × 10^−2^	0.10	8.09 × 10^−2^	7.24× 10^−2^	**0.24**
**F_6_**	**SSA**	**GOOSE**	**GWO**	**HHO**	**BKA**	**HBA**	**DBO**	**CPO**	**BTCPO**
min	7.54 × 10^−17^	1.46 × 10^−3^	2.61 × 10^−5^	8.74 × 10^−8^	6.30 × 10^−5^	2.04 × 10^−9^	2.56	4.50 × 10^−12^	**1.23 × 10^−7^**
std	8.57 × 10^−13^	7.38 × 10^−4^	0.35	9.52 × 10^−5^	1.62	1.57 × 10^−7^	0.34	8.76 × 10^−11^	**3.65 × 10^−6^**
avg	3.61 × 10^−13^	2.78 × 10^−3^	0.69	4.14 × 10^−5^	1.02	1.04 × 10^−7^	3.32	1.09 × 10^−10^	**1.48 × 10^−6^**
median	1.44 × 10^−14^	2.74 × 10^−3^	0.74	1.15 × 10^−5^	0.56	5.29 × 10^−8^	3.38	8.27 × 10^−11^	**5.13 × 10^−7^**
worse	3.53 × 10^−12^	4.87 × 10^−3^	1.48	5.06 × 10^−4^	6.42	7.88 × 10^−7^	4.00	3.12 × 10^−10^	**2.03 × 10^−5^**
time	7.26 × 10^−2^	4.65 × 10^−2^	0.10	7.40 × 10^−2^	5.23 × 10^−2^	9.21 × 10^−2^	6.36 × 10^−2^	6.09 × 10^−2^	**0.22**
**F_7_**	**SSA**	**GOOSE**	**GWO**	**HHO**	**BKA**	**HBA**	**DBO**	**CPO**	**BTCPO**
min	6.12 × 10^−6^	2.44 × 10^−2^	3.31 × 10^−4^	4.49 × 10^−6^	1.76 × 10^−5^	1.35 × 10^−5^	5.09 × 10^−5^	7.69 × 10^−5^	**7.78 × 10^−6^**
std	2.93 × 10^−4^	3.27 × 10^−2^	3.53 × 10^−4^	1.20 × 10^−4^	2.67 × 10^−4^	1.46 × 10^−4^	4.07 × 10^−4^	6.00 × 10^−4^	**4.71 × 10^−5^**
avg	3.61 × 10^−4^	6.82 × 10^−2^	8.02 × 10^−4^	1.01 × 10^−4^	1.80 × 10^−4^	1.81 × 10^−4^	5.35 × 10^−4^	9.36 × 10^−4^	**6.63 × 10^−5^**
median	2.88 × 10^−4^	6.53 × 10^−2^	7.26 × 10^−4^	6.58 × 10^−5^	7.89 × 10^−5^	1.56 × 10^−4^	3.96 × 10^−4^	7.37 × 10^−4^	**6.46 × 10^−5^**
worse	1.07 × 10^−3^	0.16	1.71 × 10^−3^	5.78 × 10^−4^	1.31 × 10^−3^	6.57 × 10^−4^	1.69 × 10^−3^	2.32 × 10^−3^	**1.91 × 10^−4^**
time	0.21	0.17	0.22	0.32	0.27	0.21	0.18	0.15	**0.49**
**F_8_**	**SSA**	**GOOSE**	**GWO**	**HHO**	**BKA**	**HBA**	**DBO**	**CPO**	**BTCPO**
min	−9.03 × 10^3^	−8.72E×10^3^	−8.03E×10^3^	−1.26 × 10^4^	−1.17 × 10^4^	−1.14 × 10^4^	−1.22 × 10^4^	−1.26 × 10^4^	**−1.26 × 10^4^**
std	5.13 × 10^2^	7.34 × 10^2^	7.06 × 10^2^	5.68 × 10^2^	1.63 × 10^3^	1.36 × 10^3^	1.79 × 10^3^	2.96 × 10^2^	**6.82 × 10^2^**
avg	−8.05 × 10^3^	−7.24 × 10^3^	−6.25 × 10^3^	−1.24 × 10^4^	−8.88 × 10^3^	−9.01 × 10^3^	−7.54 × 10^3^	−1.21 × 10^4^	**−1.16 × 10^4^**
median	−8.08 × 10^3^	−7.23 × 10^3^	−6.07 × 10^3^	−1.26 × 10^4^	−8.93E+03	−9.00 × 10^3^	−7.11 × 10^3^	−1.21 × 10^4^	**−1.17 × 10^4^**
worse	−7.05 × 10^3^	−5.40 × 10^3^	−4.90 × 10^3^	−9.50 × 10^3^	−5.13 × 10^3^	−5.28 × 10^3^	−5.50 × 10^3^	−1.14 × 10^4^	**−1.02 × 10^4^**
time	0.16	7.31 × 10^−2^	0.14	0.14	0.10	0.13	0.11	9.79 × 10^−2^	**0.32**
**F_9_**	**SSA**	**GOOSE**	**GWO**	**HHO**	**BKA**	**HBA**	**DBO**	**CPO**	**BTCPO**
min	0.00	65.90	0.00	0.00	0.00	0.00	0.00	0.00	**0.00**
std	0.00	37.02	2.17	0.00	0.00	0.00	0.00	0.00	**0.00**
avg	0.00	1.50 × 10^2^	0.72	0.00	0.00	0.00	0.00	0.00	**0.00**
median	0.00	1.54 × 10^2^	0.00	0.00	0.00	0.00	0.00	0.00	**0.00**
worse	0.00	2.26 × 10^2^	8.81	0.00	0.00	0.00	0.00	0.00	**0.00**
time	0.10	7.29 × 10^−2^	0.12	0.11	6.79 × 10^−2^	0.10	7.96 × 10^−2^	5.89 × 10^−2^	**0.26**
**F_10_**	**SSA**	**GOOSE**	**GWO**	**HHO**	**BKA**	**HBA**	**DBO**	**CPO**	**BTCPO**
min	4.44 × 10^−16^	3.37 × 10^−2^	1.11 × 10^−14^	4.44 × 10^−16^	4.44 × 10^−16^	4.44 × 10^−16^	4.44 × 10^−16^	4.44 × 10^−16^	**4.44 × 10^−16^**
std	0.00	9.13	2.63 × 10^−15^	0.00	0.00	5.05	0.00	0.00	**0.00**
avg	4.44 × 10^−16^	11.92	1.56 × 10^−14^	4.44 × 10^−16^	4.44 × 10^−16^	1.33	4.44 × 10^−16^	4.44 × 10^−16^	**4.44 × 10^−16^**
median	4.44 × 10^−16^	18.92	1.47 × 10^−14^	4.44 × 10^−16^	4.44 × 10^−16^	4.44 × 10^−16^	4.44 × 10^−16^	4.44 × 10^−16^	**4.44 × 10^−16^**
worse	4.44 × 10^−16^	19.43	2.18 × 10^−14^	4.44 × 10^−16^	4.44 × 10^−16^	19.92	4.44 × 10^−16^	4.44 × 10^−16^	**4.44 × 10^−16^**
time	9.97 × 10^−2^	7.06 × 10^−2^	0.10	0.10	6.95 × 10^−2^	0.12	0.14	8.20 × 10^−2^	**0.29**
**F_11_**	**SSA**	**GOOSE**	**GWO**	**HHO**	**BKA**	**HBA**	**DBO**	**CPO**	**BTCPO**
min	0.00	9.32 × 10^−5^	0.00	0.00	0.00	0.00	0.00	0.00	**0.00**
std	0.00	1.42 × 10^2^	7.94 × 10^−3^	0.00	0.00	0.00	0.00	0.00	**0.00**
avg	0.00	1.36 × 10^2^	4.00 × 10^−3^	0.00	0.00	0.00	0.00	0.00	**0.00**
median	0.00	1.04 × 10^2^	0.00	0.00	0.00	0.00	0.00	0.00	**0.00**
worse	0.00	3.69 × 10^2^	2.84 × 10^−2^	0.00	0.00	0.00	0.00	0.00	**0.00**
time	0.13	8.09 × 10^−2^	0.15	0.15	9.78 × 10^−2^	0.13	0.11	7.08 × 10^−2^	**0.27**
**F_12_**	**SSA**	**GOOSE**	**GWO**	**HHO**	**BKA**	**HBA**	**DBO**	**CPO**	**BTCPO**
min	2.34 × 10^−18^	3.61	1.93 × 10^−2^	6.03 × 10^−9^	3.33 × 10^−6^	4.27 × 10^−10^	1.80 × 10^−2^	3.86 × 10^−13^	**2.50 × 10^−9^**
std	1.80 × 10^−13^	5.81	2.09 × 10^−2^	2.64 × 10^−6^	0.26	9.17 × 10^−8^	0.10	4.02 × 10^−12^	**1.72 × 10^−8^**
avg	4.47 × 10^−14^	11.38	4.55 × 10^−2^	2.14 × 10^−6^	0.17	4.15 × 10^−8^	0.27	3.29 × 10^−12^	**1.71 × 10^−8^**
median	5.34 × 10^−16^	9.52	3.87 × 10^−2^	8.77 × 10^−7^	2.02 × 10^−2^	5.60 × 10^−9^	0.28	2.32 × 10^−12^	**1.22 × 10^−8^**
worse	9.83 × 10^−13^	26.37	0.10	1.11 × 10^−5^	0.93	4.54 × 10^−7^	0.45	2.15 × 10^−11^	**9.53 × 10^−8^**
time	0.37	0.27	0.32	0.61	0.49	0.32	0.29	0.28	**0.65**
**F_13_**	**SSA**	**GOOSE**	**GWO**	**HHO**	**BKA**	**HBA**	**DBO**	**CPO**	**BTCPO**
min	1.34 × 10^−16^	5.20 × 10^−4^	0.21	1.07 × 10^−7^	0.39	8.57 × 10^−8^	1.14 × 10^−2^	9.63 × 10^−12^	**8.71 × 10^−8^**
std	5.16 × 10^−13^	5.27 × 10^−3^	0.18	3.09 × 10^−5^	0.47	7.66 × 10^−2^	1.19	1.00 × 10^−2^	**2.63 × 10^−2^**
avg	2.04 × 10^−13^	4.29 × 10^−3^	0.52	1.79 × 10^−5^	1.40	7.19 × 10^−2^	1.27	1.83 × 10^−3^	**9.30 × 10^−3^**
median	2.50 × 10^−14^	1.00 × 10^−3^	0.47	6.62 × 10^−6^	1.48	7.07 × 10^−2^	0.65	3.66 × 10^−11^	**1.13 × 10^−5^**
worse	2.7 × 10^−12^	1.27 × 10^−2^	0.95	1.61 × 10^−4^	2.99	0.34	2.81	5.48 × 10^−2^	**0.11**
time	0.37	0.27	0.32	0.61	0.50	0.32	0.29	0.29	**0.66**
**F_14_**	**SSA**	**GOOSE**	**GWO**	**HHO**	**BKA**	**HBA**	**DBO**	**CPO**	**BTCPO**
min	1.00	1.99	1.00	1.00	1.00	1.00	1.00	1.00	**1.00**
std	5.81	5.50	3.64	0.30	0.75	0.65	0.93	0.00	**0.89**
avg	7.23	11.72	4.14	1.10	1.20	1.30	1.86	1.00	**1.53**
median	11.71	12.19	2.98	1.00	1.00	1.00	1.53	1.00	**1.00**
worse	12.67	22.90	12.67	1.99	4.95	2.98	2.98	1.00	**2.98**
time	0.53	0.40	0.39	0.97	0.76	0.41	0.44	0.43	**0.92**
**F_15_**	**SSA**	**GOOSE**	**GWO**	**HHO**	**BKA**	**HBA**	**DBO**	**CPO**	**BTCPO**
min	3.07 × 10^−4^	3.08 × 10^−4^	3.07 × 10^−4^	3.08 × 10^−4^	3.07 × 10^−4^	3.07 × 10^−4^	3.08 × 10^−4^	3.07 × 10^−4^	3.07 × 10^−4^
std	1.19 × 10^−6^	9.76 × 10^−3^	7.60 × 10^−3^	2.35 × 10^−4^	6.10 × 10^−3^	8.37 × 10^−3^	2.35 × 10^−4^	5.50 × 10^−18^	2.57 × 10^−4^
avg	3.08 × 10^−4^	9.44 × 10^−3^	3.65 × 10^−3^	3.97 × 10^−4^	2.39 × 10^−3^	4.69 × 10^−3^	4.90 × 10^−4^	3.07 × 10^−4^	4.83 × 10^−4^
median	3.07 × 10^−4^	1.33 × 10^−3^	3.08 × 10^−4^	3.31 × 10^−4^	3.07 × 10^−4^	3.07 × 10^−4^	4.08 × 10^−4^	3.07 × 10^−4^	3.29 × 10^−4^
worse	3.14 × 10^−4^	2.04 × 10^−2^	2.04 × 10^−2^	1.28 × 10^−3^	2.04 × 10^−2^	2.26 × 10^−2^	1.23 × 10^−3^	3.07 × 10^−4^	1.22 × 10^−3^
time	8.14 × 10^−2^	4.76 × 10^−2^	3.12 × 10^−2^	6.66 × 10^−2^	5.74 × 10^−2^	7.54 × 10^−2^	7.24 × 10^−2^	6.38 × 10^−2^	0.17
**F_16_**	**SSA**	**GOOSE**	**GWO**	**HHO**	**BKA**	**HBA**	**DBO**	**CPO**	**BTCPO**
min	−1.03	−1.03	−1.03	−1.03	−1.03	−1.03	−1.03	−1.03	−1.03
std	5.83 × 10^−16^	0.62	1.46 × 10^−8^	1.61 × 10^−11^	6.39 × 10^−16^	6.32 × 10^−16^	8.91 × 10^−16^	6.78 × 10^−16^	6.65 × 10^−16^
avg	−1.03	−0.82	−1.03	−1.03	−1.03	−1.03	−1.03	−1.03	−1.03
median	−1.03	−1.03	−1.03	−1.03	−1.03	−1.03	−1.03	−1.03	−1.03
worse	−1.03	2.10	−1.03	−1.03	−1.03	−1.03	−1.03	−1.03	−1.03
time	8.26 × 10^−2^	4.53 × 10^−2^	2.64 × 10^−2^	6.87 × 10^−2^	4.79 × 10^−2^	5.74 × 10^−2^	6.77 × 10^−2^	5.46 × 10^−2^	0.16
**F_17_**	**SSA**	**GOOSE**	**GWO**	**HHO**	**BKA**	**HBA**	**DBO**	**CPO**	**BTCPO**
min	0.40	0.40	0.40	0.40	0.40	0.40	0.40	0.40	0.40
std	0.00	8.12 × 10^−11^	4.32 × 10^−5^	2.17 × 10^−6^	0.00	0.00	6.27 × 10^−6^	0.00	0.85
avg	0.40	0.40	0.40	0.40	0.40	0.40	0.40	0.40	0.55
median	0.40	0.40	0.40	0.40	0.40	0.40	0.40	0.40	0.40
worse	0.40	0.40	0.40	0.40	0.40	0.40	0.40	0.40	5.04
time	7.22 × 10^−2^	3.76 × 10^−2^	1.73 × 10^−2^	4.84 × 10^−2^	3.18 × 10^−2^	4.98 × 10^−2^	5.98 × 10^−2^	5.15 × 10^−2^	0.14
**F_18_**	**SSA**	**GOOSE**	**GWO**	**HHO**	**BKA**	**HBA**	**DBO**	**CPO**	**BTCPO**
min	3.00	3.00	3.00	2.99	3.00	3.00	3.00	3.00	2.99
std	4.93	21.45	14,79	2.22 × 10^−8^	1.53 × 10^−15^	1.61 × 10^−15^	7.95 × 10^−3^	2.07 × 10^−15^	7.14 × 10^−16^
avg	3.90	11.10	5.70	3.00	3.00	2.99	3.00	3.00	2.99
median	3.00	3.00	3.00	3.00	3.00	2.99	3.00	3.00	2.99
worse	30.00	84.00	84.00	3.00	3.00	3.00	3.04	3.00	2.99
time	7.12 × 10^−2^	3.73 × 10^−2^	1.59 × 10^−2^	4.47 × 10^−2^	2.63 × 10^−2^	4.06 × 10^−2^	5.02 × 10^−2^	4.50 × 10^−2^	0.14
**F_19_**	**SSA**	**GOOSE**	**GWO**	**HHO**	**BKA**	**HBA**	**DBO**	**CPO**	**BTCPO**
min	−3.86	−3.86	−3.86	−3.86	−3.86	−3.86	−3.86	−3.86	−3.86
std	2.51 × 10^−15^	0.14	2.21 × 10^−3^	9.55 × 10^−4^	2.51 × 10^−15^	2.99 × 10^−3^	1.71 × 10^−3^	2.71 × 10^−15^	2.71 × 10^−15^
avg	−3.86	−3.84	−3.86	−3.86	−3.86	−3.86	−3.86	−3.86	−3.86
median	−3.86	−3.86	−3.86	−3.86	−3.86	−3.86	−3.86	−3.86	−3.86
worse	−3.86	−3.09	−3.85	−3.86	−3.86	−3.86	−3.86	−3.86	−3.86
time	8.81 × 10^−2^	4.55 × 10^−2^	3.19 × 10^−2^	7.41 × 10^−2^	5.28 × 10^−2^	6.42 × 10^−2^	7.41 × 10^−2^	6.31 × 10^−2^	0.16
**F_20_**	**SSA**	**GOOSE**	**GWO**	**HHO**	**BKA**	**HBA**	**DBO**	**CPO**	**BTCPO**
min	−3.32	−3.32	−3.32	−3.32	−3.32	−3.32	−3.32	−3.32	−3.32
std	6.03 × 10^−2^	6.11 × 10^−2^	8.29 × 10^−2^	8.94 × 10^−2^	5.71 × 10^−2^	9.29 × 10^−2^	9.37 × 10^−2^	1.34 × 10^−15^	5.83 × 10^−2^
avg	−3.26	−3.26	−3.26	−3.19	−3.28	−3.26	−3.08	−3.32	−3.29
median	−3.20	−3.20	−3.32	−3.18	−3.32	−3.32	−3.08	−3.32	−3.32
worse	−3.20	−3.20	−3.02	−2.99	−3.20	−3.02	−2.84	−3.32	−3.14
time	9.11 × 10^−2^	4.92 × 10^−2^	4.20 × 10^−2^	7.77 × 10^−2^	5.89 × 10^−2^	6.52 × 10^−2^	6.81 × 10^−2^	6.29 × 10^−2^	0.16
**F_21_**	**SSA**	**GOOSE**	**GWO**	**HHO**	**BKA**	**HBA**	**DBO**	**CPO**	**BTCPO**
min	−10.15	−10.15	−10.15	−10.15	−10.15	−10.15	−10.15	−10.15	−10.15
std	1.56	3.37	1.92	1.29	2.71 × 10^−7^	2.49	1.93	6.96 × 10^−15^	6.90 × 10^−15^
avg	−9.64	−5.64	−9.31	−5.39	−10.15	−9.34	−5.96	−10.15	−10.15
median	−10.15	−5.06	−10.15	−5.06	−10.15	−10.15	−5.06	−10.15	−10.15
worse	−5.06	−2.63	−5.06	−5.05	−10.15	−0.88	−5.06	−10.15	−10.15
time	8.26 × 10^−2^	4.60 × 10^−2^	3.35 × 10^−2^	7.45 × 10^−2^	5.63 × 10^−2^	5.98 × 10^−2^	6.65 × 10^−2^	6.86 × 10^−2^	0.15
**F_22_**	**SSA**	**GOOSE**	**GWO**	**HHO**	**BKA**	**HBA**	**DBO**	**CPO**	**BTCPO**
min	−10.40	−10.40	−10.40	−10.30	−10.40	−10.40	−10.40	−10.40	−10.40
std	1.62	3.07	3.18 × 10^−4^	0.95	5.00 × 10^−5^	3.29	2.39	1.35	1.04 × 10^−15^
avg	−9.87	−4.69	−10.40	−5.26	−10.40	−8.46	−6.51	−10.05	−10.40
median	−10.40	−3.25	−10.40	−5.09	−10.40	−10.40	−5.09	−10.40	−10.40
worse	−5.09	−1.84	−10.40	−5.08	−10.40	−1.84	−5.09	−5.09	−10.40
time	8.83 × 10^−2^	5.05 × 10^−2^	3.84 × 10^−2^	8.57 × 10^−2^	6.44 × 10^−2^	6.46 × 10^−2^	7.17 × 10^−2^	7.42 × 10^−2^	0.16
**F_23_**	**SSA**	**GOOSE**	**GWO**	**HHO**	**BKA**	**HBA**	**DBO**	**CPO**	**BTCPO**
min	−10.54	−10.54	−10.54	−5.13	−10.54	−10.54	−10.54	−10.54	−10.54
std	1.65	3.66	2.76 × 10^−4^	4.62 × 10^−3^	0.431	2.57	2.51	1.98 × 10^−15^	2.06 × 10^−15^
avg	−10.00	−5.11	−10.54	−5.13	−10.54	−9.55	−6.75	−10.54	−10.54
median	−10.54	−2.87	−10.54	−5.13	−10.54	−10.54	−5.13	−10.54	−10.54
worse	−5.13	−1.68	−10.54	−5.10	−8.17	−2.42	−5.13	−10.54	−10.54
time	9.76 × 10^−2^	5.75 × 10^−2^	4.60 × 10^−2^	0.10	7.93 × 10^−2^	7.23 × 10^−2^	7.94 × 10^−2^	8.46 × 10^−2^	0.18

**Table 3:** Comparison of Optimization Results for F_1_–F_10_.

**F_1_**	**SSA**	**GOOSE**	**GWO**	**HHO**	**BKA**	**HBA**	**DBO**	**CPO**	**BTCPO**
min	1.68 × 10^−301^	6.88	4.67 × 10^−117^	7.80 × 10^−213^	2.13 × 10^−201^	0.00	0.00	1.02 × 10^−119^	**0.00**
std	2.10 × 10^−52^	2.75 × 10^3^	1.95 × 10^−110^	0.00	4.94 × 10^−160^	0.00	0.00	2.62 × 10^−88^	**0.00**
avg	3.86 × 10^−53^	1.91 × 10^3^	3.93 × 10^−111^	5.03 × 10^−178^	9.02 × 10^−161^	1.70 × 10^−315^	0.00	4.80 × 10^−89^	**0.00**
median	1.84 × 10^−86^	5.57 × 10^2^	9.11 × 10^−114^	1.84 × 10^−192^	2.97 × 10^−194^	0.00	0.00	2.29 × 10^−100^	**0.00**
worse	1.15 × 10^−51^	9.15 × 10^3^	1.07 × 10^−109^	1.42 × 10^−176^	2.70 × 10^−159^	5.09 × 10^−314^	0.00	1.43 × 10^−87^	**0.00**
time	0.12	6.97 × 10^−2^	7.44 × 10^−2^	9.94 × 10^−2^	9.19 × 10^−2^	0.10	0.10	8.03 × 10^−2^	**0.24**
**F_2_**	**SSA**	**GOOSE**	**GWO**	**HHO**	**BKA**	**HBA**	**DBO**	**CPO**	**BTCPO**
min	0.00	3.90 × 10^−5^	0.00	0.00	0.00	0.00	0.00	0.00	**0.00**
std	1.66 × 10^−13^	7.53 × 10^2^	3.12	0.00	0.00	0.00	0.00	0.00	**0.00**
avg	3.03 × 10^−14^	8.63 × 10^2^	1.19	0.00	0.00	0.00	0.00	0.00	**0.00**
median	0.00	9.57 × 10^2^	9.09 × 10^−13^	0.00	0.00	0.00	0.00	0.00	**0.00**
worse	9.09 × 10^−13^	2.30 × 10^3^	16.23	0.00	0.00	0.00	0.00	0.00	**0.00**
time	0.11	7.11 × 10^−2^	7.18 × 10^−2^	0.13	9.95 × 10^−2^	9.22 × 10^−2^	9.35 × 10^−2^	8.02 × 10^−2^	**0.20**
**F_3_**	**SSA**	**GOOSE**	**GWO**	**HHO**	**BKA**	**HBA**	**DBO**	**CPO**	**BTCPO**
min	0.00	1.00	0.00	0.00	0.00	0.00	0.00	0.00	**0.00**
std	0.00	2.25 × 10^2^	14.75	0.00	0.00	0.00	0.00	0.00	**0.00**
avg	0.00	2.74 × 10^2^	25.91	0.00	0.00	0.00	0.00	0.00	**0.00**
median	0.00	3.32 × 10^2^	26.89	0.00	0.00	0.00	0.00	0.00	**0.00**
worse	0.00	6.26 × 10^2^	60.32	0.00	0.00	0.00	0.00	0.00	**0.00**
time	0.11	6.77 × 10^−2^	6.93 × 10^−2^	0.13	9.79 × 10^−2^	9.06 × 10^−2^	8.90 × 10^−2^	7.96 × 10^−2^	**0.21**
**F_4_**	**SSA**	**GOOSE**	**GWO**	**HHO**	**BKA**	**HBA**	**DBO**	**CPO**	**BTCPO**
min	0.00	5.00 × 10^−2^	0.00	0.00	0.00	0.00	0.00	0.00	**0.00**
std	0.00	8.25	0.66	0.00	0.00	0.00	0.00	0.00	**0.00**
avg	0.00	9.03	0.52	0.00	0.00	0.00	0.00	0.00	**0.00**
median	0.00	9.87	0.28	0.00	0.00	0.00	0.00	0.00	**0.00**
worse	0.00	24.34	2.39	0.00	0.00	0.00	0.00	0.00	**0.00**
time	0.11	6.61 × 10^−2^	6.82 × 10^−2^	0.13	9.56 × 10^−2^	9.01 × 10^−2^	8.84 × 10^−2^	7.48 × 10^−2^	**0.20**
**F_5_**	**SSA**	**GOOSE**	**GWO**	**HHO**	**BKA**	**HBA**	**DBO**	**CPO**	**BTCPO**
min	1.48 × 10^−323^	54.75	9.25 × 10^−39^	5.12 × 10^−202^	1.80 × 10^−102^	8.03 × 10^−297^	0.00	1.07 × 10^−52^	**0.00**
std	7.54 × 10^−17^	2.45 × 10^3^	0.48	0.00	4.83 × 10^−25^	8.28 × 10^−45^	4.90 × 10^−29^	1.08 × 10^−27^	**0.00**
avg	2.51 × 10^−17^	3.31 × 10^3^	0.198	2.95 × 10^−171^	1.18 × 10^−25^	1.51 × 10^−45^	1.35 × 10^−29^	2.19 × 10^−28^	**0.00**
median	2.00 × 10^−35^	2.81 × 10^3^	3.75 × 10^−32^	1.84 × 10^−190^	9.87 × 10^−42^	1.34 × 10^−118^	0.00	3.21 × 10^−30^	**0.00**
worse	3.94 × 10^−16^	7.75 × 10^3^	2.13	8.83 × 10^−270^	2.55 × 10^−24^	4.53 × 10^−44^	2.17 × 10^−28^	5.93 × 10^−27^	**0.00**
Time	0.10	6.50 × 10^−2^	6.63 × 10^−2^	9.18 × 10^−2^	9.04 × 10^−2^	8.78 × 10^−2^	8.71 × 10^−2^	8.11 × 10^−2^	**0.20**
**F_6_**	**SSA**	**GOOSE**	**GWO**	**HHO**	**BKA**	**HBA**	**DBO**	**CPO**	**BTCPO**
min	0.00	1.67 × 10^−2^	7.13 × 10^−3^	0.00	0.00	4.84 × 10^−11^	0.00	0.00	**0.00**
std	5.01 × 10^−8^	2.51 × 10^2^	1.12	6.46 × 10^−5^	7.46 × 10^−7^	9.48 × 10^−5^	4.24 × 10^−22^	5.64 × 10^−22^	**0.00**
avg	1.44 × 10^−8^	2.10 × 10^2^	0.56	2.00 × 10^−5^	1.55 × 10^−7^	4.81 × 10^−5^	7.75 × 10^−23^	1.03 × 10^−22^	**0.00**
median	8.94 × 10^−29^	35.67	4.27 × 10^−2^	1.58 × 10^−9^	9.01 × 10^−26^	5.40 × 10^−6^	0.00	0.00	**0.00**
worse	2.72 × 10^−7^	8.15 × 10^2^	4.68	3.04 × 10^−4^	4.09 × 10^−6^	3.98 × 10^−4^	2.32 × 10^−21^	3.09 × 10^−21^	**0.00**
Time	0.10	6.39 × 10^−2^	6.58 × 10^−2^	0.12	9.03 × 10^−2^	8.59 × 10^−2^	8.66 × 10^−2^	7.52 × 10^−2^	**0.20**
**F_7_**	**SSA**	**GOOSE**	**GWO**	**HHO**	**BKA**	**HBA**	**DBO**	**CPO**	**BTCPO**
min	0.00	3.28	5.38 × 10^−4^	7.19 × 10^−214^	3.03 × 10^−128^	1.79 × 10^−9^	0.00	0.00	**0.00**
std	4.25 × 10^−6^	2.32 × 10^3^	0.36	2.77 × 10^−6^	2.81 × 10^−6^	8.73 × 10^−4^	0.00	8.04 × 10^−8^	**0.00**
avg	8.09 × 10^−7^	2.95 × 10^3^	0.14	5.41 × 10^−7^	5.40 × 10^−9^	1.84 × 10^−4^	4.04 × 10^−296^	1.91 × 10^−8^	**0.00**
median	6.97 × 10^−18^	2.60 × 10^3^	1.54 × 10^−2^	3.22 × 10^−13^	2.47 × 10^−35^	8.73 × 10^−6^	0.00	4.85 × 10^−25^	**0.00**
worse	2.33 × 10^−5^	7.48 × 10^−3^	1.41	1.52 × 10^−5^	1.54 × 10^−7^	4.80 × 10^−3^	1.21 × 10^−294^	4.18 × 10^−7^	**0.00**
time	0.10	6.46 × 10^−2^	6.44 × 10^−2^	0.10	8.82 × 10^−2^	8.56 × 10^−2^	8.57 × 10^−2^	8.02 × 10^−2^	**0.20**
**F_8_**	**SSA**	**GOOSE**	**GWO**	**HHO**	**BKA**	**HBA**	**DBO**	**CPO**	**BTCPO**
min	0.00	1.54 × 10^−4^	0.00	0.00	0.00	0.00	0.00	0.00	**0.00**
std	0.00	6.53 × 10^2^	0.00	0.00	0.00	0.00	0.00	0.00	**0.00**
avg	0.00	8.71 × 10^2^	0.00	0.00	0.00	0.00	0.00	0.00	**0.00**
median	0.00	1.07 × 10^3^	0.00	0.00	0.00	0.00	0.00	0.00	**0.00**
worse	0.00	2.03 × 10^3^	0.00	0.00	0.00	0.00	0.00	0.00	**0.00**
time	0.16	0.10	0.10	0.21	0.17	0.13	0.13	0.11	**0.27**
**F_9_**	**SSA**	**GOOSE**	**GWO**	**HHO**	**BKA**	**HBA**	**DBO**	**CPO**	**BTCPO**
min	0.00	5.33 × 10^−3^	8.88 × 10^−15^	2.60 × 10^−213^	3.57 × 10^−209^	1.40 × 10^−315^	1.39 × 10^−315^	4.26 × 10^−136^	**1.39 × 10^−315^**
std	1.05 × 10^−65^	43.25	0.00	0.00	1.93 × 10^−145^	0.00	0.00	1.62 × 10^−15^	**0.00**
avg	1.95 × 10^−66^	44.25	8.88 × 10^−15^	6.17 × 10^−186^	3.52 × 10^−146^	3.31 × 10^−315^	1.53 × 10^−315^	2.96 × 10^−16^	**1.57 × 10^−315^**
median	1.06 × 10^−87^	66.77	8.88 × 10^−15^	2.04 × 10^−198^	1.05 × 10^−200^	1.60 × 10^−315^	1.45 × 10^−315^	1.71 × 10^−111^	**1.39 × 10^−315^**
worse	5.78 × 10^−65^	93.91	8.88 × 10^−15^	1.60 × 10^−184^	1.06 × 10^−144^	3.15 × 10^−314^	2.74 × 10^−315^	8.88 × 10^−15^	**6.45 × 10^−315^**
time	0.16	0.12	0.12	0.19	0.18	0.14	0.14	0.13	**0.42**
**F_10_**	**SSA**	**GOOSE**	**GWO**	**HHO**	**BKA**	**HBA**	**DBO**	**CPO**	**BTCPO**
min	0.00	2.62 × 10^−2^	3.70 × 10^−3^	9.41 × 10^−205^	7.46 × 10^−203^	1.75 × 10^−4^	1.11 × 10^−315^	0.00	**1.11 × 10^−315^**
std	1.75 × 10^−11^	56.72	17.93	5.17 × 10^−4^	1.45 × 10^−121^	2.26 × 10^−3^	0.00	3.16 × 10^−70^	**0.00**
avg	3.59 × 10^−12^	63.46	52.16	2.52 × 10^−4^	2.65 × 10^−122^	1.37 × 10^−3^	1.12 × 10^−315^	5.78 × 10^−71^	**1.11 × 10^−315^**
median	6.82 × 10^−47^	77.90	49.83	3.68 × 10^−5^	1.07 × 10^−180^	8.50 × 10^−4^	1.12 × 10^−315^	5.27 × 10^−104^	**1.11 × 10^−315^**
worse	9.61 × 10^−11^	1.59 × 10^2^	78.94	2.21 × 10^−3^	7.95 × 10^−121^	1.27 × 10^−2^	1.21 × 10^−315^	1.73 × 10^−69^	**1.11 × 10^−315^**
Time	0.15	9.72 × 10^−2^	9.86 × 10^−2^	0.19	0.15	0.12	0.12	0.11	**0.39**

**Table 4:** Comprehensive Analysis of Algorithms Comparison.

Algorithm	Convergence Precision	Stability	Time Efficiency	Application Scenarios
BTCPO	**★★★★★**	**★★★★★**	**★★★**	High-precision requirements
HHO	**★★★★**	**★★★★**	**★★★★**	Precision-efficiency balanced tasks
BKA	**★★★**	**★★★★**	**★★★★**	General optimization problems
CPO	**★★**	**★★★**	**★★★★★**	Real-time optimization
SSA	**★★**	**★★**	**★★★★**	Simple function optimization
GWO	**★**	**★**	**★★★★★**	Low-dimensional fast optimization
GOOSE	**★**	**★**	**★★★★★**	Time-sensitive applications
HBA	**★**	**★★**	**★★★★**	Function-specific optimization
DBO	**★**	**★★★**	**★★★★**	Stability-critical scenarios
PSO	**★**	**★★**	**★★★★★**	Control System Tuning
ABC	**★★**	**★★**	**★★★★**	Data Clustering and Feature Selection
CS	**★★★**	**★★**	**★★★★**	Global Optimization

**Table 5:** Comparative Results of Cantilever beam design Optimization.

Cantilever Beam	SSA	GOOSE	GWO	HHO	BKA	HBA	DBO	CPO	BTCPO
best	1.33999	1.33998	1.33997	1.34042	1.33996	1.33996	1.34005	1.33996	**1.33638**
worst	1.34149	2.29651	1.34032	1.35014	1.69676	1.34000	1.34105	1.34001	**1.34094**
std	0.00044	0.42851	0.00009	0.00249	0.11282	0.00001	0.00030	0.00002	**0.00031**
mean	1.34038	1.66006	1.34011	1.34457	1.37567	1.33997	1.34049	1.33998	**1.33843**
median	1.34022	1.38819	1.34009	1.34463	1.34000	1.33996	1.34045	1.33998	**1.33947**
time	0.1694	0.1107	0.1009	0.2864	0.2093	0.1281	0.1333	0.1241	**0.2714**

**Table 6:** Comparison Table of Three-Bar Truss Design Optimization Results.

Three-Bar Truss	SSA	GOOSE	GWO	HHO	BKA	HBA	DBO	CPO	BTCPO
best	263.8959	263.8959	263.8963	263.8959	263.8958	263.8959	263.9045	263.8958	**263.8861**
worst	263.9167	263.9017	263.9668	264.8880	263.8963	263.8966	264.1367	263.8958	**263.9487**
std	0.0068	0.0017	0.0217	0.3051	0.0002	0.0002	0.0682	0.0000	**0.0203**
mean	263.9010	263.8972	263.9107	264.0484	263.8960	263.8961	263.9584	263.8958	**263.9089**
median	263.8988	263.8967	263.9009	263.9362	263.8959	263.8960	263.9307	263.8958	**263.8978**
time	0.1525	0.0926	0.0740	0.2214	0.1640	0.1023	0.1076	0.1046	**0.2315**

**Table 7:** Comparative Results of Welded Beam Design Optimization.

Welded Beam	SSA	GOOSE	GWO	HHO	BKA	HBA	DBO	CPO	BTCPO
best	1.6705	1.7062	1.6714	1.7319	1.6713	1.6702	1.7029	1.6704	**1.6702**
worst	2.8517	2.2185	1.6816	2.2129	1.6741	1.6922	1.9857	1.6739	**1.7607**
std	0.4235	0.1421	0.0029	0.1490	0.0009	0.0085	0.1120	0.0011	**0.0330**
mean	1.8902	1.9541	1.6745	1.9070	1.6725	1.6751	1.8294	1.6711	**1.6972**
median	1.6716	1.9199	1.6737	1.8500	1.6725	1.6709	1.8125	1.6708	**1.6852**
time	0.1694	0.1107	0.1009	0.2864	0.2093	0.1281	0.1333	0.1241	**0.2714**

**Table 8:** Comparative Results of Reinforced Concrete Beam Optimization.

Test Value	SSA	GOOSE	GWO	HHO	BKA	HBA	DBO	CPO	BTCPO
best	158.8050	158.8055	158.8163	158.8165	158.8050	158.8050	159.2591	158.8050	**158.8010**
worst	158.8050	182.7366	158.8817	159.2114	158.8341	158.8050	164.1658	158.8050	**158.8069**
std	0.0000	8.1121	0.0210	0.1479	0.0109	0.0000	1.5201	0.0000	**0.0006**
mean	158.8050	164.9084	158.8438	159.0049	158.8182	158.8050	159.8407	158.8050	**158.8052**
median	158.8050	162.4429	158.8427	158.9946	158.8186	158.8050	159.3697	158.8050	**158.8050**
time	0.0768	0.0501	0.0373	0.1013	0.0860	0.0735	0.0789	0.0595	**0.1415**

## Data Availability

The data that support the findings of this study are available from the corresponding author upon request. There are no restrictions on data availability.
